# Solubilization Behavior of Polyene Antibiotics in Nanomicellar System: Insights from Molecular Dynamics Simulation of the Amphotericin B and Nystatin Interactions with Polysorbate 80

**DOI:** 10.3390/molecules21010006

**Published:** 2015-12-24

**Authors:** Meysam Mobasheri, Hossein Attar, Seyed Mehdi Rezayat Sorkhabadi, Ali Khamesipour, Mahmoud Reza Jaafari

**Affiliations:** 1 Department of Chemical Engineering, Science and Research Branch, Islamic Azad University, Tehran 1477893855, Iran; attar.h@srbiau.ac.ir; 2 Tofigh Daru Research and Engineering Company (TODACO), Tehran 1397116359, Iran; 3 Department of Medical Nanotechnology, School of Advanced Technologies in Medicine, Tehran University of Medical Sciences, Tehran 1417755469, Iran; rezayat@sina.tums.ac.ir; 4 Department of Toxicology and Pharmacology, Pharmaceutical Sciences Branch, Islamic Azad University, Tehran 193956466, Iran; 5 Center for Research and Training in Skin Diseases and Leprosy, Tehran University of Medical Sciences, Tehran 1416613675, Iran; khamesipour@tums.ac.ir; 6 Biotechnology Research Center, Nanotechnology Research Center, School of Pharmacy, Mashhad University of Medical Sciences, P. O. Box: 91775-1365, Mashhad 917751365, Iran

**Keywords:** polyene antibiotics, Amphotericin B, Nystatin, Polysorbate 80, solubilization, molecular dynamics simulation, drug formulation, drug delivery systems, nanomedicine

## Abstract

Amphotericin B (AmB) and Nystatin (Nys) are the drugs of choice for treatment of systemic and superficial mycotic infections, respectively, with their full clinical potential unrealized due to the lack of high therapeutic index formulations for their solubilized delivery. In the present study, using a coarse-grained (CG) molecular dynamics (MD) simulation approach, we investigated the interaction of AmB and Nys with Polysorbate 80 (P80) to gain insight into the behavior of these polyene antibiotics (PAs) in nanomicellar solution and derive potential implications for their formulation development. While the encapsulation process was predominantly governed by hydrophobic forces, the dynamics, hydration, localization, orientation, and solvation of PAs in the micelle were largely controlled by hydrophilic interactions. Simulation results rationalized the experimentally observed capability of P80 in solubilizing PAs by indicating (i) the dominant kinetics of drugs encapsulation over self-association; (ii) significantly lower hydration of the drugs at encapsulated state compared with aggregated state; (iii) monomeric solubilization of the drugs; (iv) contribution of drug-micelle interactions to the solubilization; (v) suppressed diffusivity of the encapsulated drugs; (vi) high loading capacity of the micelle; and (vii) the structural robustness of the micelle against drug loading. Supported from the experimental data, our simulations determined the preferred location of PAs to be the core-shell interface at the relatively shallow depth of 75% of micelle radius. Deeper penetration of PAs was impeded by the synergistic effects of (i) limited diffusion of water; and (ii) perpendicular orientation of these drug molecules with respect to the micelle radius. PAs were solvated almost exclusively in the aqueous poly-oxyethylene (POE) medium due to the distance-related lack of interaction with the core, explaining the documented insensitivity of Nys solubilization to drug-core compatibility in detergent micelles. Based on the obtained results, the dearth of water at interior sites of micelle and the large lateral occupation space of PAs lead to shallow insertion, broad radial distribution, and lack of core interactions of the amphiphilic drugs. Hence, controlled promotion of micelle permeability and optimization of chain crowding in palisade layer may help to achieve more efficient solubilization of the PAs.

## 1. Introduction

The therapeutic index (TI) of many surface active drugs is limited due to their tendency to self-associate in aqueous medium, resulting in low solubility, limited bioavailability, and potential severe side effects of these pharmaceuticals [1,2]. The clinical advantages of overcoming this challenge have provoked a large body of research into the development of formulations with improved pharmaceutical properties [3]. Among the most surface active drugs with an extending therapeutic use is the Amphotericin B (AmB). With a long history of clinical application, this polyene antibiotic (PA) remains the “gold standard” therapy against systemic mycotic infection, among all reasons, due to its broad-spectrum fungicidal activity, superior pharmacokinetic and pharmacodynamic profile compared to the related agents, and low rate of resistance [4,5]. Featured as well with leishmanicidal activity, AmB is also increasingly used as the drug of choice for treatment of visceral leishmaniasis in the cases of resistance against antimonials [6,7,8]. In spite of this striking therapeutic potency, the full potential efficacy of this drug is not realized in clinical practice due to its diverse dose-dependent side effects ranging from irreversible renal failure to central nervous system and liver damage to infusion-related reactions such as chills, fever, hypotension, dyspnea, rigors, arthralgias, nausea, vomiting and headaches [9]. Another amphiphilic PA with close structural similarity with AmB [10] is Nystatin (Nys), a potent antimycotic agent widely used for treatment of superficial mycoses [11,12]. While Nys has shown broad-spectrum fungicidal activity [11,13,14], and effectiveness against some fungal infections resistant to AmB [13,14], its potential for systemic antifungal therapy has remained untapped because of high toxicity of the drug when administered intravenously [11,12,15,16].

Extensive evidence has linked the toxicity of AmB and Nys to their presence in the aggregated state [17,18,19,20]. The mechanism of action of these PAs is based on the ability of their monomers to selectively promote permeability of ergostrol-rich fungal membranes rather than cholesterol-containing mammalian membranes, leading to leakage of ions and small molecules essential for fungal cell life, and ultimately lysis and death of the pathogen [21,22,23,24]. At the aggregated state, however, these drugs lose their selective recognition of the membranes and attack the membranes of both fungal and mammalian cells, causing cytotoxicity [17,18,19,20,21,22,23,24,25,26]. The solubilized delivery of PAs at monomeric state, hence, has been proposed as a possible pathway to their higher TI [9,27,28,29].

A powerful biomimetic strategy for enhancing the solubility of amphiphilic drugs is to incorporate them in the micellar assemblies of surface-active agents (surfactants) [2,30,31]. Owing to their particular structure which limits presence of water in the internal sites, micelles provide an energetically more favorable environment for residence of amphiphilic drugs compared with the bulk aqueous solution [32]. The advantages associated with micellar delivery systems, including ease of development, affordable costs, well-studied mechanisms of delivery, and the large parameter space for engineering specific properties, retains them as attractive vehicles for solubilized drug delivery. The Fungizone^®^, the major commercially available formulation of AmB produced and marketed by Bristol-Myers Squibb [28], is a dispersion of AmB in micellar solution of deoxycholate [1,33,34]. There have also been attempts to develop alternative micellar/vesicular formulations of this antibiotic using various excipients [1,35]. Similar efforts have been made for solubilized delivery of Nys, exemplified by liposomal [15], polymeric micellar [29], and more recently surfactant-based niosomal [36,37] delivery systems, all of which aimed at enabling parenteral administration of the drug. Although these formulations have proven great promise for improving the solubility and alleviating the toxicity of PAs, their widespread use in clinical practice is subject to more breakthrough advances in the field. In this respect, rational design of novel delivery systems inspired by the detailed knowledge of drug-excipient interactions can promote the efficiency of the research.

Rational development of pharmaceutical formulations is being increasingly aided by *in silico* methods [38,39,40,41]. Specifically, molecular dynamics (MD) simulation has proven powerful in providing molecular-level insight into drug-expedient interactions, particularly in those aspects not able to be readily explored by routine experimental techniques [39,41,42]. There are intriguing success stories of MD simulations use in drug formulation research, having provided insights into the location and mode of distribution of drug molecules in the vehicles [42], morphology of drug-loaded particles [43], aggregation behavior and mechanism of toxicity of drugs in presence of excipients [44], mechanisms, driving forces, and loading efficiency of drug encapsulation in carriers [45,46], and efficiency of particular formulations for solubilized drug delivery [46,47]. These studies have attracted attention to the MD simulations as a promising approach to optimal engineering of drug delivery systems [38,39,41,47].

In relation to PAs, MD simulations have been extensively used in the conformational analysis of the AmB in aqueous [48,49,50] and lipidic [51,52] media, characterizing the molecular aspects of AmB-biomembranes interactions [50,53,54,55,56,57], elucidating the mechanism of action of the drug [58,59], and understanding the nature of relationship between its molecular organization and selective toxicity [58,60,61,62]. Despite, however, MD simulations are not applied thus far in exploring the factors contributing to the solubilization of PAs, in contrast for instance to anticancer drugs such as paclitaxel [46,63]. AmB and Nys are structurally characterized by a rectangular glycosylated lactone ring, with a hydrophobic conjugated polyene chain on one edge, a hydrophilic polyhydroxyl chain on the opposite edge, a polar head comprised of carboxylate anion linked to the main ring, and an ammonium cation attached to the mycosamine moiety [48,64]. This structure renders these drug molecules as both amphiphilic and amphoteric, leading to their poor solubility, limited permeability/bioavailability and complex aggregation behavior. The same molecular characteristics also underlie the difficulties associated with developing formulations of AmB and Nys with desired profile [1,9]. This situation indicates a rationale for the use of *in silico* methods to further explore the molecular peculiarities of AmB and Nys in solubilized state, and identify factors influencing their solubilization efficiency.

Following our previous work on developing liposomal preparation of AmB [65], in this study, we sought to explore the utility of MD simulation in providing information about the of PA-carrier interactions. Our aim is to gain an understanding of the behavior of PAs in micellar systems and derive potential implications for future formulation developments. We selected Polysorbate 80 (P80) as the model surfactant. P80 is a non-toxic non-ionic detergent widely used for solubilizing and emulsifying hydrophobic pharmaceuticals, cosmetics, and food additives [30,31,32]. The P80 micelles have already been shown to be capable of remarkably enhancing Nys solubility in the aqueous medium [27]. P80 has also been used as a surfactant/co-surfactant in various formulations of AmB, such as microemulsions [33,66], nanoemulsions [67,68], and SEDDS [69,70]. Therefore, the MD simulation study of interactions of PAs with P80 may also provide insight for improving the efficiency of the currently available formulations of these drugs.

Among the barriers to the direct use of MD simulation to drug formulation practice are the associated computational demand and time-inefficiency, which may offset its potential advantages such as reduced experimental costs and labor. The extent of the challenge, however, is increasingly narrowing by the advent of efficient molecular modeling techniques such as coarse-grained (CG) modeling, allowing for less computational cost while maintaining certain levels of accuracy. In the CG modeling, the individual atoms are grouped together according to certain criteria to form CG representative interaction sites (beads), and the MD calculations are then performed between these interaction sites rather than between the individual atoms. This technique results in remarkable improvement of computational efficiency, thereby possibility of monitoring molecular interactions in a broad range of time and scale, which is essential for understanding many time-depending molecular-level phenomena. One of the standardized approaches to CG MD simulations is the MARTINI method developed and extended by Marrink *et al.* [71,72]. This method enables regular mapping of the atomic structure of a particular molecule to a CG molecular model followed by systematic model parameterization. MARTINI model has proven accurate in reproducing structural, dynamic, and thermodynamic properties for a broad range of systems and state points in accord with experimental data [73]. In this research, we used MARTINI approach to study the molecular dynamics of the interactions between PAs and P80. The study focuses on the aggregation of the drugs and the surfactant in aqueous medium, encapsulation behaviors of PAs, the intermolecular interactions involved in the drug encapsulation processes, the structural properties of P80-PA nanomicelles, and the stability of drug-surfactant mixed micellar system. The implications of the results for drug formulation development are discussed.

## 2. Results and Discussion

### 2.1. Simulation of P80 Micelle

#### 2.1.1. Simulation of Single P80 Molecule in Aqueous Medium

For characterization of P80-PA interactions is the aqueous medium, it is first essential to gain a description of single and multiple molecular behaviors of each substance, separately. Simulation of a single P80 molecule was carried out by solvating it in 3500 CG water particles (0.5 w%), and running the production for 150 ns (Table 1, Simulation 1). Assuming that the mode of P80 hydration would influence its interaction with PAs, this simulation was carried out to characterize structuring of water around the surfactant molecule. As the radial distribution function (RDF) against water and hydration numbers of P80 moieties (Supplementary Material 1, Figure S1) indicates the hydration pattern represents the expected contrast in the density of water around the hydrophilic and hydrophobic domains.

#### 2.1.2. Formation and Characteristics of P80 Micelle

**Figure 1:**
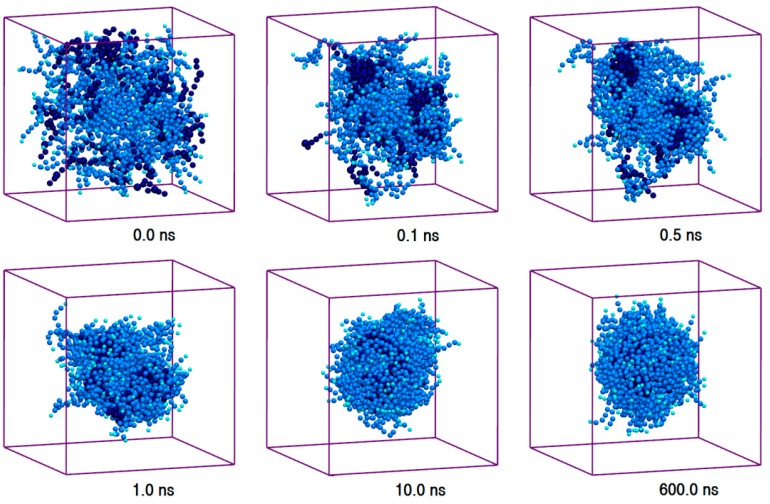
Trajectory snapshots of micellation of Polysorbate 80 (P80) in the aqueous medium at different simulation times (water particles have been removed for clarity).

We studied the self-assembly and properties of P80 micelle in the aqueous medium. A system of 10 w% P80 in water was configured by randomly distributing 60 P80 molecules in 9817 CG water particles, and the production simulation was run for 600 ns (Table 1, Simulation 2). The selected trajectory snapshots of P80 self-assembly are illustrated in Figure 1, and the corresponding animation is provided in Supplementary Materials 2, Video S1. As can be seen, the randomly dispersed surfactant molecules (*t* = 0) self-assemble to two distinct clusters within 0.1 ns. The individual clusters become more packed during the next 0.3 ns. The clusters are then fused to each other (*t* = 1.0 ns) to minimize water-hydrocarbon contact, leading to the formation of a micelle containing all individual surfactant molecules (P80_60_). The plot of energy evolution (Supplementary Materials 1, Figure S2a) shows that the micelle reaches equilibrium state within around 3.0 ns, and remains energetically stable until the end of simulation.

**Table 1:** Summary of the study simulations.

Study Phase	Simulation	No.	Time (ns)	No. of Molecule(s)				Concentration (w%)			Ratio
				P80	AmB	Nys	Water Particles	P80	AmB	Nys	PA-to-P80
1: Simulation of P80	Single P80 molecule	1	150	1	-	-	3500	0.5	-	-	-
		2	600	60	-	-	9817	10	-	-	-
		3	600	100	-	-	16,361	10	-	-	-
		4	600	150	-	-	24,542	10	-	-	-
		5	600	60	-	-	4363	20	-	-	-
	Multiple P80 molecules	6	600	60	-	-	2545	30	-	-	-
		7	600	60	-	-	1636	40	-	-	-
		8	600	200	-	-	32,723	10	-	-	-
		9	600	200	-	-	14,544	20	-	-	-
		10	600	200	-	-	8484	30	-	-	-
2: Simulation of PAs	Single AmB	11	150	-	1		410		3	-	
	Multiple AmB	12	150	-	9		11024	-	1	-	-
	Single Nys	13	150	-	-	1	410		-	3	
	Multiple Nys	14	150	-	-	9	11024	-	-	1	-
3: Simulation of P80-PA systems	Single AmB and single P80	15	150	1	1	-	410	4.1	2.9	-	0.7
	Single AmB encapsulation	16	150	60	1	-	9817	10	0.1	-	0.01
	Multiple AmB encapsulation	17	600	60	9	-	9817	10	1	-	0.1
		18	600	60	17	-	9600	10	2	-	0.2
		19	600	60	26	-	9485	10	3	-	0.3
		20	600	60	34	-	9382	10	4	-	0.4
		21	600	60	43	-	9817	10	5	-	0.5
		22	600	100	9	-	16,153	10	0.7	-	0.06
		23	600	150	9	-	24,426	10	0.4	-	0.04
		24	600	200	29	-	32,351	10	1	-	0.1
		25	600	200	14	-	14,364	20	1	-	0.05
		26	600	200	9	-	8368	30	1	-	0.03
	Single Nys and single P80	27	150	1	-	1	410	4.1	-	2.9	0.7
	Single Nys encapsulation	28	150	60	-	1	9817	10	-	0.1	0.01
	Multiple Nys encapsulation	29	600	60	-	9	9817	10	-	1	0.1
		30	600	60	-	17	9600	10	-	2	0.2
		31	600	60	-	26	9485	10	-	3	0.3
		32	600	60	-	34	9382	10	-	4	0.4
		33	600	60	-	43	9817	10	-	5	0.5
		34	600	100	-	9	16,153	10	-	0.7	0.06
		35	600	150	-	9	24,426	10	-	0.4	0.04
		36	600	200	-	29	32,351	10	-	1	0.1
		37	600	200	-	14	14,364	20	-	1	0.5
		38	600	200	-	9	8368	30	-	1	0.3

**Figure 2:**
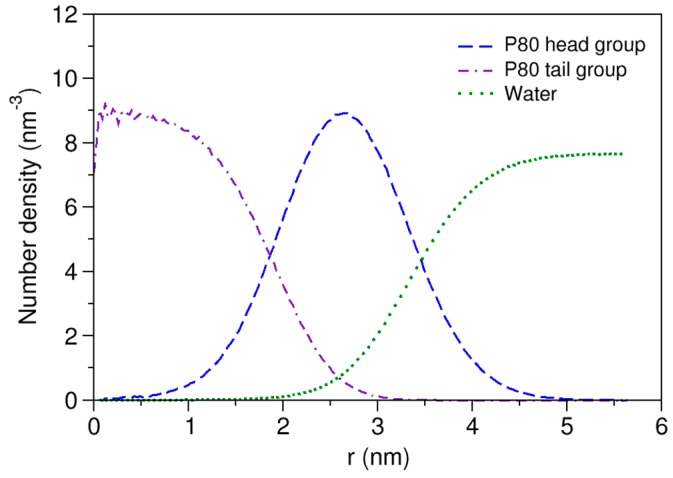
Density distribution of water and head and tail of P80 from micelle center of mass.

The time evolution of radius of gyration (*R_g_*) is plotted in Supplementary Materials 1, Figure S2b. The average *R_g_* during the last 500 ns of the simulation was calculated to be 27.5 Å, which shows excellent match with the experimentally determined value of 27.2 Å [74], being obtained more accurately compared with the corresponding value in the CG simulation study of Amani *et al.* (26.2 Å) [75]. The *R_g_* of the micelle core was 16.9 Å. Using the formula Rm=53 Rg [76], these values give an effective micelle radius of 35.5 Å, with the micelle core radius (*R_c_*) of 21.8 Å and the shell thickness (ST) of 13.7 Å. The shape of the P80 micelle was estimated based on the principal moments of inertia (PMI). The ratio of the average principal axes of inertia (PAI) was calculated to be 1.31:1.14:1.00 over the last 500 ns of the simulation. The eccentricity (e) of micelle was determined using the formula $e=\sqrt{1-\frac{c^{2}}{a^{2}}}$, where *c* and *a* are the shortest and the longest computed semi-axes, respectively. The eccentricity value was obtained 0.65 which along with PAI ratios reveals the semi-spherical (ellipsoidal) shape of the micelle. The SASA of micelle and the hydrophilic % were estimated to be 556.1 nm^2^ and 82%, respectively. Figure 2 displays the radial density functions for head and water from the micelle COM. As seen, the head group peaks around 26 Å and the water penetrates up to 17 Å from the COM.

The micelle parameters including *R_g_*, eccentricity, SASA, hydrophilic %, and head peak and water penetration radii are in good agreement with the corresponding values reported from atomic-scale simulation of the Tween 80 micelle of canonical structure with equal length of POE head branches at *N_ag_* of 60 (27.4 Å, 0.63, 540 nm^2^, 87.6%, 22 Å, 16 Å, respectively) [77], demonstrating the comparable accuracy of MARTINI model with atomic-scale simulation in representing our surfactant micellar system. Following the validation of model, we characterized some more properties of the P80 micelle, as described in details Supplementary Materials 1. In short, the micelle structure shows little dependence on *N_ag_*, micelle hydrocarbon moieties are poorly hydrated (Supplementary Materials 1, Figure S3) consistent with observation from the density profile, and the calculated lateral diffusivity of the micelle (2.3 × 10^−7^ cm^2^/s^−1^), approaches well to the experimentally reported value of 3.0 × 10^−7^ cm^2^/s^−1^ [78].

#### 2.1.3. Effect of Surfactant Concentration on Micelle Size and Morphology

Experimental and *in silico* studies have shown that the surfactant micellar assemblies typically undergo size and morphological transition in response to the altered concentration [79,80,81]. The geometrical properties of surfactant carriers may impact their drug loading capacity and delivery efficiency [63,82]. We investigated the potential effect of surfactant concentration on the structural properties the P80 micelle, and the effect of system size and initial configuration on the aggregation behavior of the surfactant. Briefly, a sphere-to-cylinder shape transition (Supplementary Materials 1, Figure S4) and an increased size (Supplementary Materials 1, Figure S5) of the micelles with concentration augment were observed. These observations are consistent with the concentration-dependent structural transition of the non-ionic surfactants reported in previous experimental and *in silico* studies [79,80,83], supporting the validity of our modeling. In addition, the size of the largest micelle formed at various system sizes and initial configurations ranged around the value of 60 verifying both the assumed aggregation number of P80 and the robustness of the simulated micellar system against system size, at least within the simulation timeframe. More detailed description of the relationship between structural properties of P80 micelle and the concentration and the effect of system size on simulation results is provided in Supplementary Materials 1.

### 2.2. Simulation of AmB and Nys

#### 2.2.1. Simulation of a Single PA Molecule in Water

**Figure 3:**
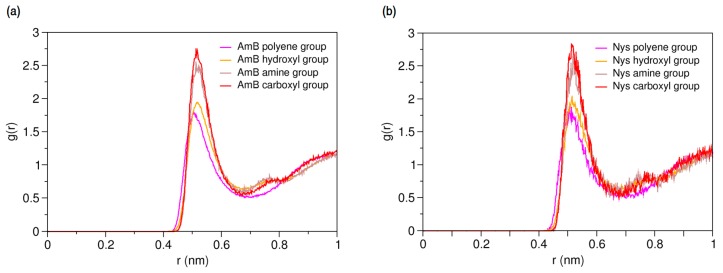
Average radial pair distribution functions for various moieties of (**a**) a single Amphotericin B (AmB) molecule; and (**b**) a single Nystatin (Nys) molecule against water particles.

To characterize hydration and dynamics of PAs in water, a single molecule of AmB and Nys was simulated in presence of 400 CG water particle (3 w%) separately, for 150-ns (Table 1, Simulations 11 and 13, respectively). Figure 3a,b present the RDFs for various moieties of the AmB and Nys against water, respectively. As seen, each of the PA moieties is surrounded by a hydration shell with different degrees of water structuring. The average number of water particles within the hydration shell around AmB (Nys) moieties was calculated to be 8.02 (8.13) for the carboxyl group, 7.69 (7.75) for the amine group, 4.52 (4.28) for the hydroxyl group, and 4.22 (4.22) for the polyene group, which is compliant with the expected order. The higher hydration intensity of the charged moieties (carboxyl and amine groups) reflects their comparatively strong tendency towards the polar solvent. However, one would expect to see a larger difference in hydration number between the hydroxyl and polyene groups given their hydrophilic and hydrophobic nature, respectively, which is not captured by the coarse-grained modeling, in contrast to the atomic-scale simulation [48]. The average distance between the polyol and polyene chains in water for both drug molecules was calculated to be 4.73 ± 0.07 Å, which is nearly equal to the van der Waals radius of the CG water particle. The average distance between the most distant sites on the lactone ring (beads 8 and 15) was calculated to be 14.9 ± 0.13 Å for both drug molecules.

The 3-dimensional and lateral diffusion constants of AmB were calculated to be 3.1 × 10^−6^ cm^2^/s^−1^ and 4.6 × 10^−6^ cm^2^/s^−1^, respectively. The corresponding coefficients for Nys were estimated to be 3.4 × 10^−6^ cm^2^/s^−1^ and 4.9 × 10^−6^ cm^2^/s^−1^. Although the lack of parallel experimental data does not allow comparison, the calculated diffusion constants can be used to compare the dynamics of PAs before and after entrapment in P80 micelle, as will be discussed below.

#### 2.2.2. Aggregation of PAs in the Aqueous Solution

**Figure 4:**
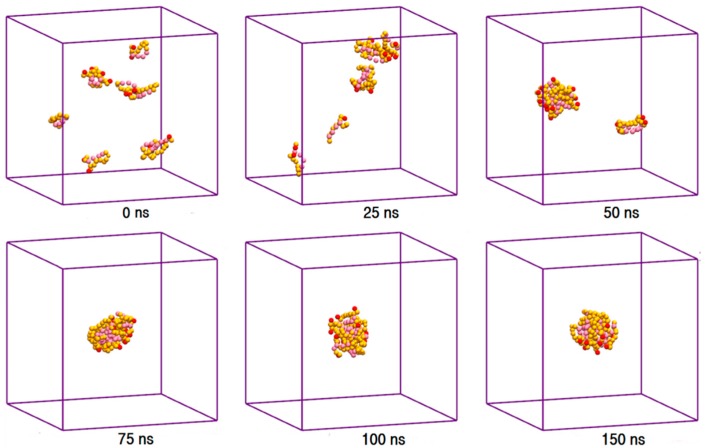
Trajectory snapshots of aggregation of AmB in the aqueous medium at different simulation times (water particles have been removed for clarity).

The toxic side effects of PAs are largely related to their presence in the aggregated form [17,18,19]. To monitor the drug self-association effect in the AmB-W and Nys-W solutions we extended the simulations of single PA molecule in water to an aqueous solution of 9 PA molecules at 1 w% (Table 1, Simulations 12 and 14, respectively). To consider the possible effect of initial system configuration on aggregation behavior, each simulation was carried out for 10 times at different random initial configurations. The selected trajectory snapshots from sample simulations of AmB and Nys in water are given in Figure 4 and Supplementary Materials 1, Figure S6, respectively. The trajectories show that self-association of PAs in water proceeds initially by formation of small oligomeric clusters, followed by association of these aggregates to form a large oligomeric assembly.

**Figure 5:**
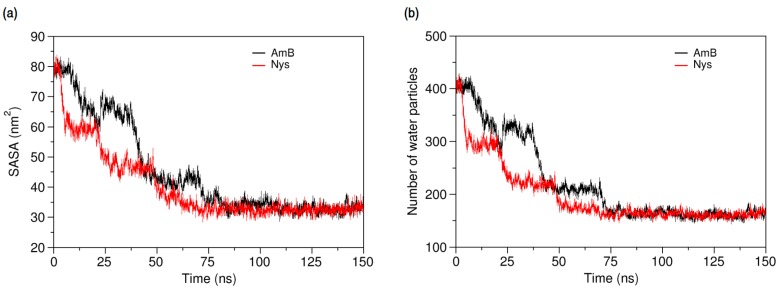
Comparison of the time-evolution of (**a**) solvent accessible surface area (SASA) of AmB and Nys; and (**b**) average number of water particles around AmB and Nys molecules within 7 Å, during self-association process.

Aggregation of hydrophobic molecules in the aqueous media is known to be driven by unfavorable contacts between water and their hydrophobic domains [1,3,84]. It has been shown that as the self-association proceeds, the solvent accessible surface area (SASA) of the hydrophobic solute is constantly limited in a linear correlation with the unfavorable contacts [46,84,85]. Thereby, the SASA can be used for semi-quantitatively tracking the self-aggregation of non-soluble agents in the aqueous solvent. To quantify the progress of AmB and Nys self-association (1 w%), we calculated their SASA and the average number of water particles around each PA molecule within the first hydration shell (7 Å) over the simulation period. Figure 5a presents the SASA profile of PAs and Figure 5b shows average number of water particles around PA molecules observed from a sample simulation of 9 molecules of each drug at identical initial configurations. The SASA profiles exhibit declining trend with the simulation time, indicating that the self-aggregation proceeds by the tendency of PAs to protect their hydrophobic moieties from water.

This is confirmed by the progressive decrease of average number of water particles around each PA molecule (Figure 5b) as SASA becomes increasingly limited. The time for evolution of SASA of AmB (Nys) to equilibrium in 10 simulations at various initial configurations ranged from 75 ns (70 ns) to 210 ns (185 ns) and averaged at 127 ± 52 ns (113 ± 44 ns). The pattern of SASA variations indicates the stepwise formation of larger aggregates from the smaller ones, as also observed from the trajectories. The formation of the intermediate and final aggregates proceeds marginally faster for Nys compared with AmB. Although self-aggregation kinetics is sensitive to the initial system setup, considering the identical initial configuration of both AmB-W and Nys-W systems, the slightly faster aggregation of Nys may reflect its observed higher diffusivity.

Supplementary Materials 1 Figure S7 illustrates the variation of PA-PA and PA-W interaction energies during aggregation process, observed from the above-mentioned sample simulations. As seen, the interaction energy of each of PAs with itself and water falls and increases, respectively, to equilibrium level, suggesting gradual replacement of PA-W interactions by PA-PA interactions along the aggregation process. Despite the absence of hydrogen bond interactions which are cardinal to the hydrophobic effect [1,3,84], the above data confirm that CG simulations can mimic this effect sufficiently enough to capture the behavior of amphiphiles in aqueous medium.

### 2.3. Simulation of P80-PA Systems

#### 2.3.1. Interaction of PAs with a Single P80 Molecule

**Figure 6:**
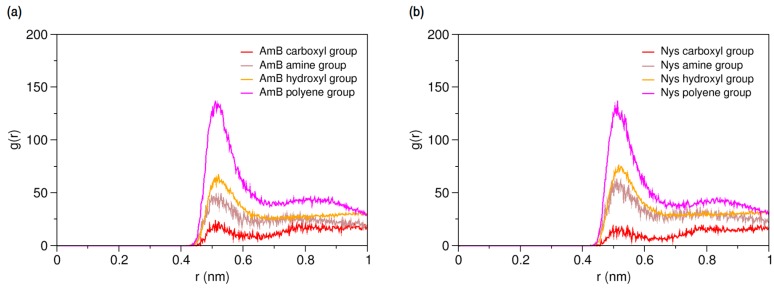
Average radial pair distribution functions for various moieties of (**a**) AmB; and (**b**) Nys against single P80 molecule in aqueous medium.

The main purpose of this study was to characterize the interactions AmB and Nys with P80 in aqueous environment. This investigation was initiated by simulating the interaction of a single molecule of each drug in presence of a single P80 molecule in water (Table 1, Simulations 15 and 27, respectively). Radial pair distribution functions between various moieties of P80 and PAs can give a detailed account of drug-surfactant interactions. From RDFs for molecular groups of AmB and Nys against P80 (Figure 6a,b, respectively) it is seen that the polyene groups of PAs tend to have by far the highest level of interaction with the surfactant, compared with other moieties. Accordingly, the chromophores of PAs are preliminarily responsible for driving the interaction of these antibiotics with P80. The carboxyl group shows a little tendency to interact with the detergent, which is due to its preferred affinity for the aqueous surrounding (Figure 4).

**Figure 7:**
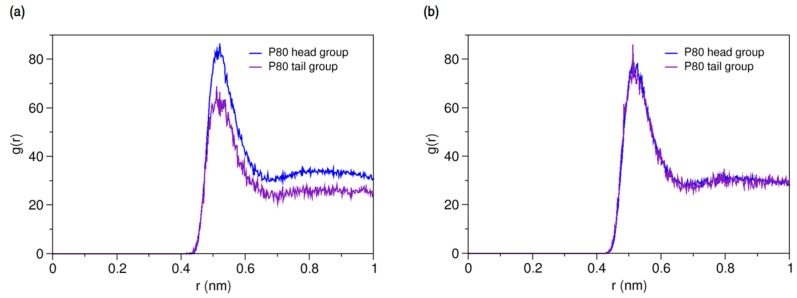
Average radial pair distribution functions for head and tail of P80 against (**a**) AmB; and (**b**) Nys in aqueous medium.

On the other hands, the RDFs of P80 head and tail against AmB and Nys (Figure 7a,b, respectively) reveals the comparable level of interactions of head and tail with PAs. Particularly, head and tail groups show very similar radial distribution pattern around Nys molecule. This is surprising given the larger population of head group interactions sites and the stronger affinity of drug molecules for the surfactant head group compared with the tail. This observation may in part be due to the lower density of the polar solvent nearby the hydrophobic alkyl chain, which would allow for fewer unfavorable interactions of PAs with water while interacting with the tail. Further possible explanation for this observation is the preferred integration of PAs with the branch 1 (beads 7–11) of the POE chain among all polyethylene oxide branches of P80, as described in Supplementary Materials 1 (Figure S8).

#### 2.3.2. Encapsulation of PAs into P80 Micelle

**Figure 8:**
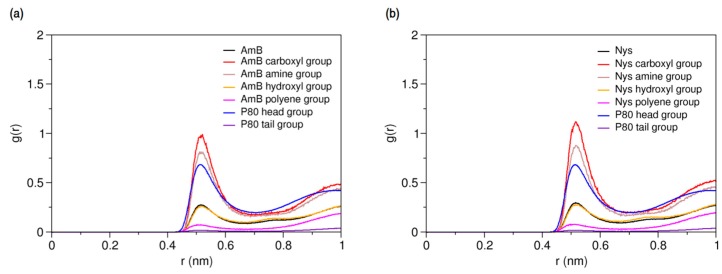
Average radial pair distribution functions for different molecular groups of (**a**) P80-AmB mixed micelle; and (**b**) P80-Nys mixed micelle against water particles.

Solubilizing capability of surfactants is largely dependent on their presence in micellar conformation [32,86,87,88,89]. We simulated the interaction of PAs with P80 micelle to explore the solubilization behaviors of these drugs, and properties of the mixed micelles form. Firstly, the interaction of a single PA molecule with a pre-formed equilibrium micelle of 60 P80 molecules (P80_60_) was simulated (Table 1, Simulations 16 and 28, respectively) in order to characterize the hydration and dynamics of the drug within the micelle. After equilibrium was reached, the presence of PAs in micelles was confirmed by the sharp peak of P80 RDFs against AmB. The RDFs for different molecular groups of P80_60_-AmB_1_ and P80_60_-Nys_1_ mixed micelles against water are illustrated in Figure 8a,b, respectively.

The hydration level of PAs in the micelle falls in-between that of P80 head and tail groups. In comparison with the free state (Figure 5), the hydration of the polyol and polyene chains of PAs in the micelle is significantly limited. However, the charged groups of PAs are more strongly hydrated than the surfactant head. The above figure represents a competition between hydrophobic and hydrophilic moieties of PAs to modulate the interactions of the encapsulated drugs with water, leading to the dynamic residence of the PAs in micelle. To quantify the net dynamics of PAs within the micelle phase, the 3-dimentional and lateral diffusion coefficients of the entrapped drug molecules were calculated by eliminating contributions of translation of the COM and rotation of the micelle. Three-dimensional diffusion constant was estimated using Einstein equation (see Methods Section) by calculating the MSD of the remaining translational motion of PAs. For determining lateral diffusion constant, the MSD was calculated for projection of PAs displacements on the micelle spherical surface. The 3-dimensional and lateral diffusion constants for AmB were estimated 3.01 × 10 ^−7^ cm^2^s^−1^ and 4.61 × 10^−7^, respectively, whereas the corresponding values for Nys were obtained 3.06 × 10^−7^ cm^2^s^−1^ and 4.38 × 10^−7^. Comparison of these values with the respective values for free PA molecules in water (see Section 2.2.1) shows an order-of-magnitude decline in the freedom of motion of the PAs at encapsulated state. Such a suppression of dynamics, though not the only influencing factor, is important to the residence time of drugs in the micelle and prevention of rapid release [63,90,91]. We also calculated the average distance between the polyol and polyene chains of the encapsulated AmB (Nys) to be 4.79 ± 0.06 Å (4.78 ± 0.04 Å) and the average distance between the most distant sites on the macrolide ring (beads 8 and 15) to be 15.2 ± 0.11 Å (15.3 ± 0.11 Å). Both values are significantly larger than the corresponding values for free single AmB (Nys) molecule in water (*p* < 0.01), indicating that the structure of PAs is stretched in the P80 micelle. The attenuated effect of water repulsive forces and the augmented effect of surfactant attractive forces on the encapsulated drugs may be the reason.

**Figure 9:**
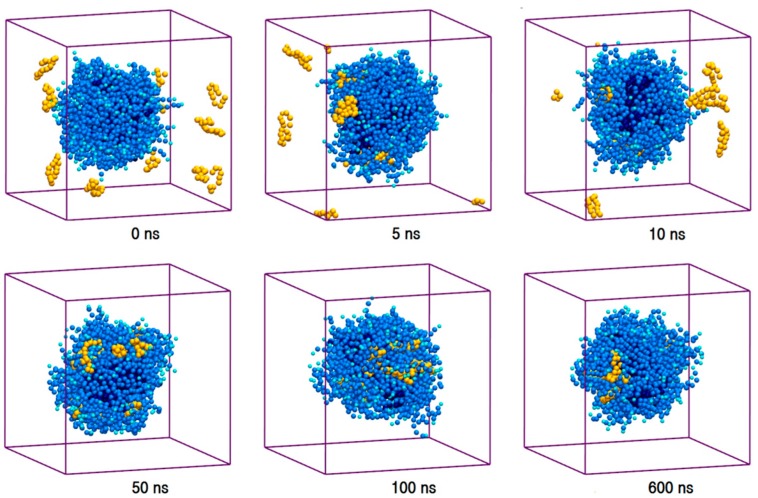
The trajectory snapshots of encapsulation of AmB molecules into P80 micelle at different simulation times (water particles have been removed for clarity).

The properties of pharmaceutical formulations emerge from the collective interaction of the drug molecules with the excipients [1,2,3,28,30,32,63]. To explore the collective interaction of AmB and Nys with P80 micelle, for each PA, a simulation setup consisted of 9 drug molecules in presence of 60 P80 molecules (1 w% of PA, 10 w% of P80, and drug-to-surfactant ratio of 0.1) was configured and run (Table 1, Simulations 17 and 27, respectively). To take into account the possible effect of initial system configuration, the simulation was carried for 10 times at different random initial configurations. Selected snapshots of the trajectories from sample simulations of P80_60_-AmB_9_-W and P80_60_-Nys_9_-W systems are presented in Figure 9 and Supplementary Materials 1, Figure S9, and the corresponding animations can be viewed from Supplementary Materials 2, Videos S2 and S3, respectively. The trajectories show a spontaneous and relatively rapid migration of PA monomers from the bulk towards the micelle phase; the time for incorporation of all AmB (Nys) molecules into the micelle ranged from 13 ns (10 ns) to 64 ns (60 ns) in 10 simulations. The average time for encapsulation of AmB (Nys) over 10 simulations was calculated to be 38.5 ± 18.4 ns (35.2 ± 16.4 ns) which is significantly (*p* < 0.01) lower than the average time for aggregation of the drug molecules (127 ± 51.8 ns (113 ± 44 ns)) at the same concentration.

**Figure 10:**
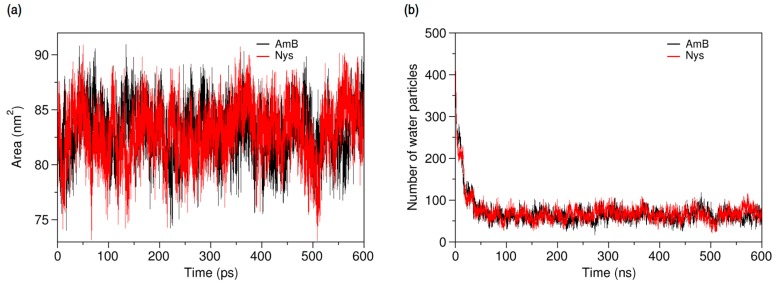
(**a**) Solvent accessible surface area of AmB and Nys molecules; and (**b**) average number of water particles around AmB and Nys molecules within 7 Å, during and after encapsulation into the P80 micelle.

The dominance of encapsulation kinetics over aggregation kinetics is crucial for inhibiting drug aggregation and achieving high drug loading efficiency [92]. Therefore, the observed faster speed of PAs encapsulation than that of their aggregation, at the same drug concentration, indicates the potential of P80 to solubilize PAs in water. Profiling SASA of AmB and Nys over simulation time (Figure 10a) reveals that SASA of AmB (Nys) significantly increases (*p* < 0.05) from the 80.85 ± 2.33 nm^2^ (80.91 ± 2.42 nm^2^) corresponding to free monomeric state of 9 drug molecules in water to 83.02 ± 2.27 nm^2^ (83.05 ± 2.49 nm^2^) corresponding to the encapsulated state of the drug. The SASA increase would be due to the aforementioned expanded conformation of PAs in the micelle. Such an increase in SASA represents the capability of P80 for monomeric solubilization of PAs.

Figure 10b compares the changes in the average number of water particles surrounding AmB and Nys molecules, within 7 Å. As can be seen, the number of water particles around PAs dramatically decreases during the encapsulation of the drugs. From comparison of Figure 5b and Figure 10b, it is revealed that the number of water particles around the AmB (62.13) and Nys (62.12) in the micelle is lower (by approximately 62%) than that in their respective aggregates (162.8 and 162.6, respectively). Hence, the encapsulated state of PAs allows significantly more water protection for the drugs than their aggregated state. This finding together with faster kinetics of PAs encapsulation than aggregation and the monomeric solubilization of PAs in the micelle consistently reflects the experimentally documented capability of P80 micelles to remarkably increase the aqueous solubility of Nys [27], and explains the underlying molecular-level mechanism.

**Figure 11:**
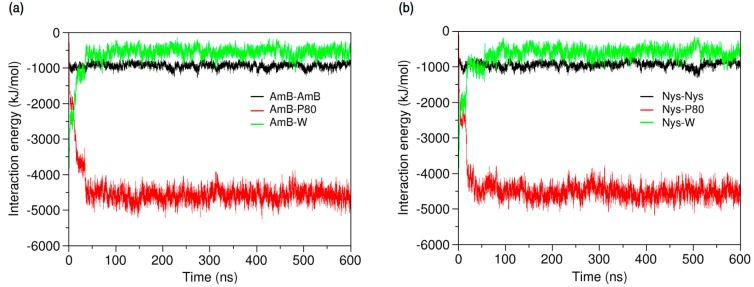
Time-evolution of average energy of interactions (**a**) between AmB and AmB, P80, and water; and (**b**) between Nys and Nys, P80, and water.

Further insight into the forces controlling the encapsulation behaviors of PAs may be obtained from analysis of interaction energies in the P80-PA-W systems. As illustrated in Figure 11a,b, respectively, the average energy of AmB-AmB and Nys-Nys interactions remains unchanged before and after encapsulation, confirming the monomeric solubilization of these drugs, already implicated from SASA profiles. The increasing energy of AmB-W and Nys-W interactions during encapsulation depicts the solubilization process as a hydrophobic phenomenon. The equilibrium energy of PA-W interactions remains below zero (~−500 KJ/mol), suggesting that PAs tend to preserve a certain level of interaction with water. Considering the diminishing availability of water in the direction of micelle COM (Figure 3), this tendency translates into the restricted insertion of PAs into the micelle (see below for further discussion). The energy profiles also show the progressive substitution of PA-P80 interactions with PA-W ones, as the encapsulation proceeds. The absolute variation in the energy of PA-P80 interactions is approximately 1000 KJ/mol larger than that of PA-W ones, indicating that PAs establish more stable interactions with the surfactant as compared with water. Therefore, the encapsulation of PAs into the P80 micelle is not only driven by the tendency of drugs to escape from water, but by their stronger interactions with P80 compare with water as well. While contribution of drug-surfactant interactions to micellar solubilization and their stabilizing effect on the incorporated solutes has been mostly reported in polymeric surfactant solutions [32,93], our observations provide evidence for this to be the case in detergent micellar systems, as well.

#### 2.3.3. Structure of P80-PA nanomicelles

Structural properties of P80_60_-AmB_9_ and P80_60_-Nys_9_ nanomicelles were investigated by analysis of the corresponding simulation trajectories. The *R_g_* of the both micelles was calculated to be 27.8 Å, which is slightly (0.3 Å) larger than that of drug-free micelle. The cores of mixed micelles have a *R_g_* of 16.9 Å which is identical to that of the drug-free micelle. Based on this data, the effective radius of both mixed micelles is determined to be 35.9 Å with a *R_c_* 21.8 Å and *ST* of 14.1 Å.

**Figure 12:**
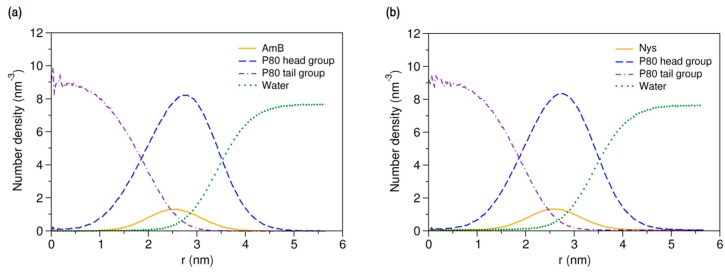
Radial density distributions for various molecular groups of (**a**) P80-AmB mixed micelle; and (**b**) P80-Nys mixed micelle.

More details on the internal structure of P80-PA nanomicelles can be obtained by analysis of the radial density of their components. The density profiles for P80_60_-AmB_9_ and P80_60_-Nys_9_ micelles are presented in Figure 12a,b, respectively. As can be seen, the entrapped PAs are partitioned between the shell and the core-shell interface of the micelle, with most of the drugs preferentially located in the interface. This observation agrees well with the experimentally reported solubilization of Nys in the core-corona interface of P80 micelles [27]. Additionally, the close density peaks of shell and PAs together with the complete surround of PAs by the shell and the broad tail of shell in the core suggests that the presence of drugs in the core-dominant region of the interface is largely mediated by the dynamics of core-shell interactions.

The average location of both PAs is determined to be 27 Å from the micelle COM. Given the abovementioned geometrical characteristics of the mixed micelles, the drugs are located on average 5.2 Å away from the core surface and 8.9 Å before the shell surface. The ratio of drug distance from the COM to the micelle radius is 0.75 which shows the relatively shallow insertion of PAs into the micelle. In addition, the density profile displays the wide radial distribution of PAs ranging from 12 to 42 Å from COM, indicating that a portion of the drug molecules frequently leave the micelle up to 6.1 Å and re-renters to it (as could also be seen from the trajectories). All these observations may be interpreted as the limited capability of P80 for stable solubilization of PAs and possible burst release or precipitation of the drugs.

Location of drugs in the matrix of pharmaceutical carriers is an important parameter in developing micellar drug delivery systems, as it affects various aspects of the formulation efficiency, including the drug loading capacity, formulation stability, and drug release behavior [39,94,95]. From the energetics points of view, the limited diffusion of PAs into the P80 micelle can be attributed to three possible factors: (1) the repulsive interactions between the drugs and the core; (2), attractive interactions between drugs and the shell; and (3) attractive interactions between drugs and water. The first hypothesis is ruled out by the findings from simulation of single-P80-single-PA interactions, where the drugs showed comparable level of interaction with both head and tail groups. Regarding the second hypothesis, the broad radial distribution of head moieties and the thickness of micelle head imply that PAs have enough room to insert deeper into the micelle and still largely interact with the corona, undermining the role of drug-surfactant interaction as a limiting factor for drug diffusion. Thereby, the affinity for water remains the only energetic factor significantly constraining radial insertion of the drugs. As such, the depth at which PAs may penetrate in the micelle will inevitably depend on the permeability of water across the micelle. The implications of this conclusion for the stability of PAs micellar formulation will be discussed in the Section 2.4.

The localization behavior of PAs can also be understood in terms of their topological properties. Molecular length, branching, size, shape, and structure have been shown to affect both preferred localization site and degree of solublization of the solubilizates [86]. Recently, from a systematic DPD simulation study, Guo *et al.* [94] observed that the insertion of large and/or linearly shaped molecules into the micelle core is more challenging as compared with that of the small and/or branched molecules, due to their lower diffusion constant and the larger space required for penetration. Given that AmB and Nys are characterized by a relatively large and linear topology, the crowding of the chains and the lack of space in the regions closer to the core can hinder deep penetration of these molecules.

**Figure 13:**
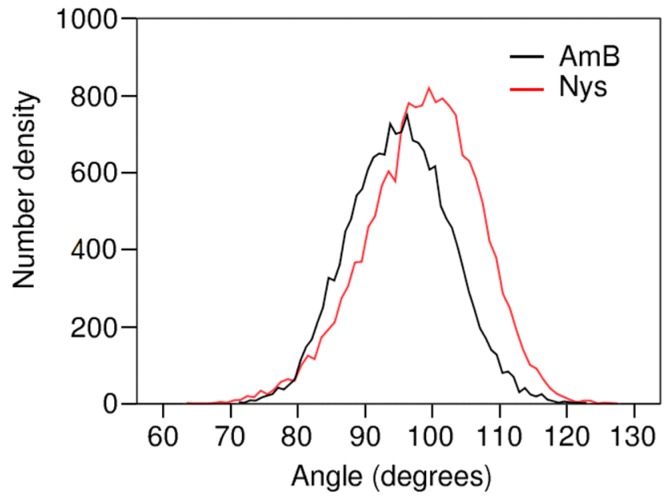
Bead4-Bead7-COM angle distribution for the encapsulated AmB and Nys.

The extent to which the topological factors may contribute to the localization behavior of AmB and Nys may depend on their orientation in the micelle. To interrogate this issue, the orientation of these PAs at encapsulated state was characterized by calculating the angle between polyene chromophore chain of each drug molecule and the micelle radius. This was done by calculating the average distribution of angles between the vector extending from micelle COM to bead 7 and the vector between the beads 7 and 4 of each drug molecule. Figure 13 compares the frequency distribution of these angles between AmB and Nys. The average of angle distribution for AmB appears at around 95°, indicating that the hydrophobic face of the drug orients virtually perpendicular to the radius of the micelle. The inclination as high as 5° toward the surface is representative of higher affinity of the carboxyl group for water compared with the hydroxyl chain. The angle distribution of Nys averages at 98°, which is marginally (3°) higher than the corresponding value for AmB. This can be explained by the closer position of the P4 particle to the carboxyl group in Nys compared with AmB, making the polar head of Nys more inclined towards the polar solvent.

The perpendicular orientation of PAs polyene face with respect to micelle radius reveals the tendency of PAs to maintain their hydrophilic and hydrophobic interactions balanced with the aqueous environment, demonstrating that the encapsulation behavior of PAs is fine-tuned by water influence. Considering the rectangular shape of PAs, the observed angular position of the drug molecules corresponds to the widest lateral space they may occupy in the micelle which is already packed along the orthogonal direction. This mode of orientation together with the hydrophilic interactions of the drugs with water hamper deep and firm residence of PAs in the micelle in a synergistic manner, further elucidating the molecular-level challenges to stable micellar solubilization AmB and Nys.

#### 2.3.4. Micellar Solvation of PAs

**Figure 14:**
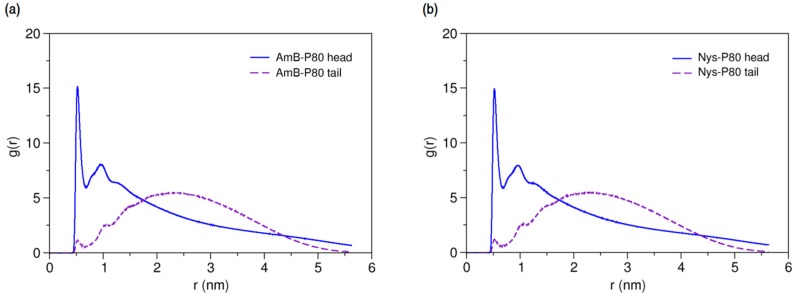
Radial distribution functions for head and tail of P80 against (**a**) AmB; and (**b**) Nys in P80_60_-AmB_9_ and P80_60_-Nys_9_ mixed micelles, respectively.

Characterizing the local environment around drug molecule in carrier using MD simulations may provide insight into solvation behavior of the drug in the excipient medium [96]. Figure 14a compares the RDFs for AmB against P80 head and tail groups at the solubilized state and the corresponding illustration for Nys is given in Figure 14b. The sharp peak of P80 hydrophilic head in these figures shows that the drug molecules are densely surrounded by the POE-chain-dominated corona. Conversely, lack of a clear peak on RDF plots of P80 hydrophobic tail is indicative of poor concentration of hydrocarbon groups around the drug molecules. The RDF analysis hence suggests that AmB and Nys are almost exclusively solvated in an aqueous POE medium provided within the micelle. The poor interaction of PAs with the P80 tail in the micelle is not due to the lack of miscibility, as it was already seen from simulation of single-PA-single-P80 systems that the AmB and Nys can interact with both POE and hydrocarbon domains at comparable levels. The lack of such interactions at encapsulated state would result rather from the distance between the preferred location of PAs and the hydrocarbon moieties concentrated in the core, preventing effective intermolecular interactions. The impact of distance becomes more apparent by comparing the *R_g_* of AmB (25.5 Å) and Nys (25.7 Å) at encapsulated state with that of micelle hydrophobic chains (16.5 Å). The average distance of PAs from the core was calculated to be 5 Å which remained steady throughout the simulation period. These observations predict limited effect of drug-core compatibility on solubilization of PAs in detergents similar to P80, due to distance related lack of drug interaction with the core. This hypothesis finds support by the work of Croy and Kwon [27] in which Nys exhibited no significant micelle/water partitioning difference between P80 and a structurally similar surfactant, the Cremophor EL, with lower core polarity compared with P80.

#### 2.3.5. Complementary Analysis of Properties of P80-PA Micellar Systems

We further analyzed the properties of P80-PA nanomicellar solution by characterizing the effect of PAs on the conformational properties of P80 micelles (Table 1, Simulations 17–21 and 29–33), the effect of drug load and micelle size on localization of PAs (Table 1, Simulations 17–21 and Simulations 29–33), the effect of PAs on the stability of P80 micellar system (Table 1, Simulations 24–26 and Simulations 36–38), pattern of PAs distribution in micellar solution (Table 1, Simulation 24), and the drug loading capacity of P80 as a carrier of PAs (Table 1, Simulations 17–21 and Simulations 29–33). Briefly, conformational analysis of P80-PA micelle indicated the structural robustness of P80 micelle against a wide range of drug load; no significant impact of drug load and micelle size on localization of the drugs was identified; the stability of P80 multi-micellar system was found to be sensitive to drug load; PAs exhibited a heterogeneous distribution among micelles; and P80 micelle showed a relatively high capacity to encapsulate PAs at monomeric state. Detailed description of the above investigations is provided in Supplementary Material 1 (Figures S10 and S11).

### 2.4. Implications for Drug Toxicity and Formulation

Experimental data have shown a significant capability of P80 as a solubilizing carrier of PAs, either in single use or in combination with other excipients [27,33,66,70]. Our simulation study both confirms and rationalizes this potential by indicating the dominant kinetics of drugs encapsulation over self-association, lower hydration of the drugs at encapsulated state compared with aggregated state, monomeric solubilization of the drugs in the micelle, stabilization of the incorporated drugs by drug-surfactant interactions, significantly reduced diffusion of the entrapped drugs, high loading capacity of the micelle, and stability of micelle structure against drug loads.

Despite these advantages, some drawbacks of P80-based solubilization of PAs were also revealed. These include limited penetration of PAs towards the core and their preferred localization at the interface, the broad distribution of drug molecules over micelle radius, orientation of PAs corresponded to the largest lateral occupation space, limited interaction of PAs with the core, and sensitivity of micellar system stability to drug introduction. Most of these disadvantages are associated with the kinetic stability of the P80-PA mixed micelles, which would influence the drug release behavior. Specifically, the residence of drugs at the corona or core-corona interface has been associated with the burst release which negatively affects delivery performance [90,97,98] and may also induce local or systemic side effects [90,99,100,101,102]. Burst release has been reported in some micellar preparations of AmB [103,104,105] and has been associated with entrapment of this drug in shell or core-shell interface [105]. Burst release of AmB and Nys is particularly important regarding the dose-dependent nature of most side-effects of these drugs, including nephrotoxicity [106]. Therefore, an effective P80-based formulation of a PAs entails complementary strategies for suppressing the envisioned premature release.

The persistent affinity of PAs for water in the P80 micelle matrix renders localization of these drugs directly dependent on the extents of water diffusion. Because the deep interior of the conventional detergent micelles is typically void of water [107,108,109], the amphiphilic drugs have to remain sufficiently close to the surface to preserve their required interactions with the aqueous solvent. This may not only limit the residence time of drug in the micelle due to the short path of outward diffusion, but may also frustrate the contribution of drug-core interactions to the solubilization, as observed in this theoretical and previous experimental studied [27], due to the distance of the preferred location of the drug from the core. These notions brings up the idea that controlled promotion of micelle permeability may help deeper penetration of the amphiphilic drugs by enabling interaction of drug with water at higher depths. This hypothesis is corroborated by the evidence that block-copolymeric micelles which have proven superior over detergents in incorporating PAs into the core [29,104,106,110,111] and controlling their aggregation state drug during release [112,113,114], also allow penetration of water up to their cores [107,115]. In this view, water permeability of micelle may be regarded as a mechanism for passive delivery of amphiphiles to the core region, whereby drug-core interaction if adequately strong, could exert its solubilizing/stabilizing effect. Further confirmation of this hypothesis in future studies may introduce new approaches to stable solubilization of PAs in conventional non-toxic detergents.

Pharmaceutical formulations are often based on combination solubilizing strategies rather than relying only on single surfactant, to realize the potential solubilizing synergies of multiple agents. A notable exception is the Fungizone^®^, in which a single detergent surfactant (SDC) is used to solubilize AmB, leading thus to the administration of the formulation being associated with dose-dependent adverse effects [9]. The study of Tancrèdef *et al.* also reported a synergistic toxic effect of AmB when administered with P80 as the only excipient [116]. Our observation *in silico* that the solubilizer-drug interactions contribute to stabilization of the drugs signifies the notion that using co-surfactants or stabilizers in detergent-based formulations of PAs may yield more control over drug release behavior, which is poor in preparations such as Fungizone^®^ [112].

Our results also revealed the significance of orientation-induced lack of lateral space for penetration of PAs. Therefore, consideration is due to the crowding of the chains in the palisade layer in formulation design for these relatively large rectagularly-shaped drug molecules. At least theoretically, both permeability enhancement and chain crowing control are procurable by tuning the surfactant hydrophobic to hydrophilic chain.

## 3. Experimental Section

### 3.1. Study Design

Although the particular objective of the present work was to investigate interaction of PAs with P80 in the aqueous medium, a detailed description of the molecular behavior of each substance in water (W) is required for interpretation of P80-PA-W systems simulation results. Therefore, a study was designed in three phases to address (i) simulation of P80; (ii) simulation of PAs; (iii) simulation of P80-PAs interactions in aqueous medium. Due to the very large computer demand otherwise, all simulations were carried out far above the critical micelle concentration (CMC) of both of the surfactant and drugs. Table 1 summarizes the simulation setups in each phase of the study.

### 3.2. Modeling of P80 and PAs

**Figure 15:**
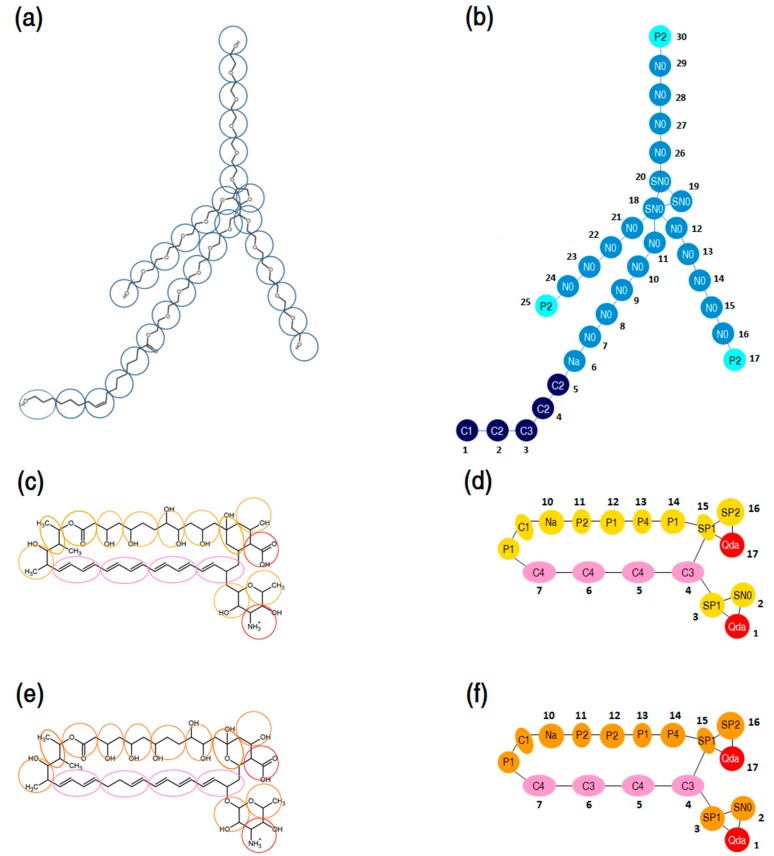
(**a**) Course-grain mapping of P80; (**b**) coarse-grain model of P80, comprising 30 interaction sites: 21 sites for the poly-oxyethylene (POE) chain, 5 for alkyl chain, and 3 for terminal polar groups and 1 for the ester group; (**c**,**e**) coarse-grain mapping of AmB and Nys, respectively; (**d**,**f**) coarse-grain model of AmB and Nys, respectively, with 17 interaction sites: 4 sites for the polyene chain, 1 for carboxyl group, 1 for amine group, and 11 for the hydroxyl and other groups.

The MARTINI coarse-graining method introduced by Marrink *et al.* [71,72] was adopted to develop CG models of P80 and PAs. The CG mapping of P80 molecular structure is presented in Figure 15a. P80 was modeled by 30 CG sites, 24 of which representing the hydrophilic head group (N- and P-type particles), and 6 representing the hydrophobic tail (C-type particles). The CG mapping of AmB and Nys molecular structures is illustrated in Figure 15b,c respectively. PAs were represented by 17 CG sites; 4 of which representing polyene chain (C-type particles), 2 for charged groups (Qda-type particle), 8 for hydroxyl groups (P-type particles) and 3 for other groups (C and N particle types). The detailed description of the CG mapping of P80 and PAs is given in the Supplementary Materials 1. The CG models of both surfactant and drug molecules were initially parameterized according to the default MARTINI force field parameters and then optimized to reproduce the structural properties of the corresponding united-atom models, including distances, angles, and dihedrals.

### 3.3. Simulation and Analysis

MD simulations and the subsequent analyses were carried out using GROMACS v. 4.6.3 simulation package [117] and the simulation trajectories were visualized using VMD [118]. The full description of simulation details and methods of analysis of the simulation trajectories are provided in the Supplementary Materials 1.

## 4. Conclusions

Molecular knowledge of drug interaction with the excipients is crucial for overcoming the challenge of amphiphilic drug solubilization. Our CG MD simulation study provided a series of relevant clues for understanding the solubilization behaviors of PAs in micellar system and their underling mechanisms. Based on the obtained results, although the encapsulation process is predominantly driven by hydrophobic forces, various aspects of PAs solubilization including dynamics, hydration, localization, orientation, and solvation are largely influenced by the drug’s hydrophilic interactions with water. Our simulations confirmed the experimentally evident capability of P80 for solubilizing PAs, and rationalized it by (i) dominant kinetics of drugs encapsulation over self-association; (ii) lower hydration of the drugs at encapsulated state compared with aggregated state; (iii) monomeric solubilization of drugs; (iv) stabilization of the incorporated drugs via drug-surfactant interactions; (v) significantly reduced diffusion of the drugs at entrapped state compared with free sate in water; (vi) high loading capacity of the micelle; and (vii) stability of micelle structure against drug overload. On the other hands, consistent with experimental data, PAs were found to preferentially localize at the core-shell interface of P80 micelle and exhibit a broad radial distribution extending beyond the micelle radius. This localization behavior was elucidated to stem from the combined effects of PAs partial affinity for water, limited micellar penetration of water, and perpendicular orientation of PAs with respect to the micelle radius. In addition, PAs were distributed heterogeneously among the micelles and the stability of multi-micellar system was found to be sensitive to the incorporation of the drugs. These data imply that in spite of high solubilizing potential of P80, stable solubilization of PAs in P80 micelle is a challenging task requiring combination approach.

The observation that encapsulation behavior of PAs is largely dependent on their interaction with water suggests that the dearth of water in interior of the P80 micelle may be a major factor restricting deep and stable encapsulation of PAs. Hence, the controlled promotion of micelle permeability may enable interaction of PAs with water at deeper sites, thereby their improved interactions with the core. Further confirmation of this hypothesis may introduce novel strategies for stable detergent-based solubilization of the amphiphilic drugs. Moreover, the particular orientation of PAs which corresponds to their largest lateral occupation space highlights the limiting effect of chain crowing on penetration of these relatively large and rectangularly-shaped drugs. Ideally, both permeability enhancement and chain crowding control are approachable by modulating the surfactant hydrophobic to hydrophilic chains.

By characterizing both advantages and drawbacks of P80 as a potential carrier of PAs, addressing a wide range of solubilization-related phenomena consistent with experimental results, and generating experimentally testable hypotheses for drug solubilization, our study demonstrates that MD simulations even at CG level can be used as an efficient explorative tool in pharmaceutical formulation engineering.

## Supplementary Materials

Supplementary File 1: Extended description of Section 2.1.1, Section 2.1.3, Section 2.3.5, Section 3.2 and Section 3.3, and Supplementary Figures S1–S11; Supplementary File 2: Simulation trajectory videos (Videos S1–S3). Supplementary materials can be accessed at: http://www.mdpi.com/1420-3049/21/1/6/s1.
