# Supplementary material for: Solubilization Behavior of Polyene Antibiotics in Nanomicellar System: Insights from Molecular Dynamics Simulation of the Amphotericin B and Nystatin Interactions with Polysorbate 80
Source: Molecules. 2015 Dec 24;21(1):6. doi: 10.3390/molecules21010006 (PMC6273564; doi:10.3390/molecules21010006)
Supplement: Supplementary file 1 [file molecules-21-00006-s001.zip › molecules-104000-supplementary-final/molecules-104000-supplementary-final.pdf]

Supplementary Materials: Solubilization Behavior of Polyene Antibiotics in Nanomicellar System: Insights from Molecular Dynamics Simulation of the Amphotericin B and Nystatin Interactions with Polysorbate 80

Meysam Mobasheri [1,*], Hossein Attar [1,2], Seyed Mehdi Rezayat Sorkhabadi [3,4], Ali Khamesipour [5] and Mahmoud Reza Jaafari [6,*]

1. Simulation of P80

1.1. Simulation of Single P80 Molecule in Aqueous Medium

Adequately present the expected contrast in the density of water around the interaction of a single P80 molecule with water was studied by performing Simulation 1 (main manuscript, Table 1) mainly to characterize the hydration pattern of the molecule. The radial pair distribution functions for each of the P80 moieties against water (Figure S1) indicate a shell of hydration around each of the P80 molecular groups with different water density. The average number of water particles within the shell around P80 molecular groups was calculated to be 30.1 for P2, 23.14 for N0 + SN0, 8.29 for Na, 6.83 for C3, 5.41 for C2, and 8.06 for C1. The hydration numbers hydrophilic and hydrophobic domains.

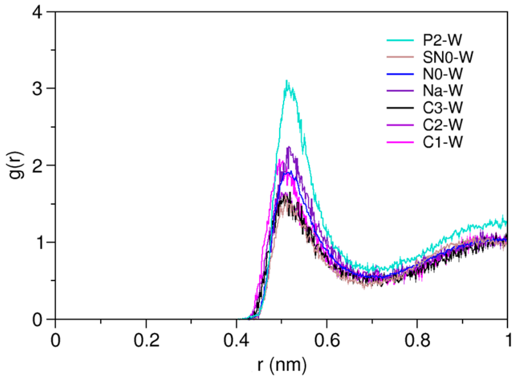

Figure S1. Average radial pair distribution functions for various molecular groups of a single P80 molecule against water particles.

1.2. Formation and Characteristics of P80 Micelle

We also calculated the micelle parameters at larger N_{ag} (100 and 150), to characterize their variations with micelle size. A linear increase of R_g (β = 0.085, R^2 = 0.97), R_c (β = 0.092, R^2 = 0.99), and ST (β = 0.035, R^2 = 0.99) was observed with N_{ag} taking values of 60, 100, and 150 (main manuscript, Table 1, Simulations 2-4). In addition, a proportional increase of R_c/R_m (β = 0.0002, R^2 = 0.99) and decrease of ST/R_m (β = −0.0002, R^2 = 0.99) with N_{ag} was recorded indicating micelle growth being accompanied by relative expansion of the core and contraction of the shell. Nonetheless, the smallness of the β values suggests that the structure of the micelle has little dependence on N_{ag}.

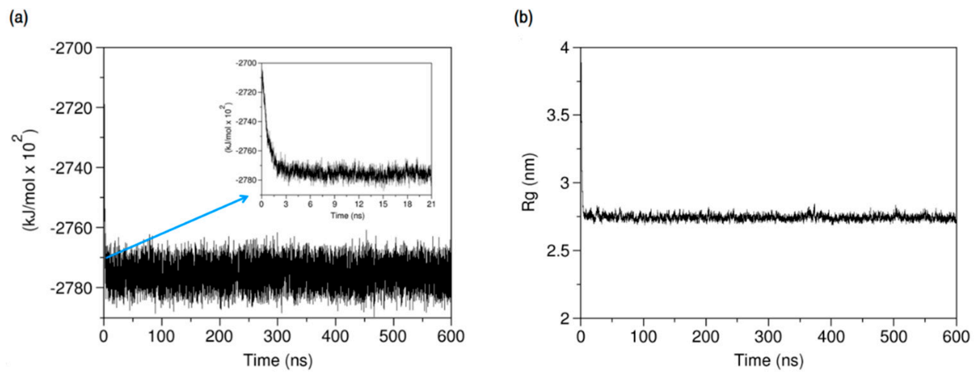

Figure S2. Time-evolution of (**a**) potential energy of the system and (**b**) radius of gyration of P80 during simulation of $P80_{60}$-W system. (The inserted figure in Figure S2a pointed by the blue arrow shows the time-evolution of the system's potential energy within the first 21 ns)

Figure S3 displays the radial distribution of different CG particles of P80 micelle with respect to the water particles. Compared with single free molecular state, in the micellar configuration the hydration level of the surfactant molecular groups has become more distinct. P2 particle type shows the highest peak indicating the stronger hydration of the particle due to its polarity, followed by SN0 and N0, all of which residing on the hydrophilic head of CG P80 molecule. C1, C2, and C3, on the other hand, show very low and inconspicuous peaks, representative of the poor hydration of the micelle hydrocarbon moieties.

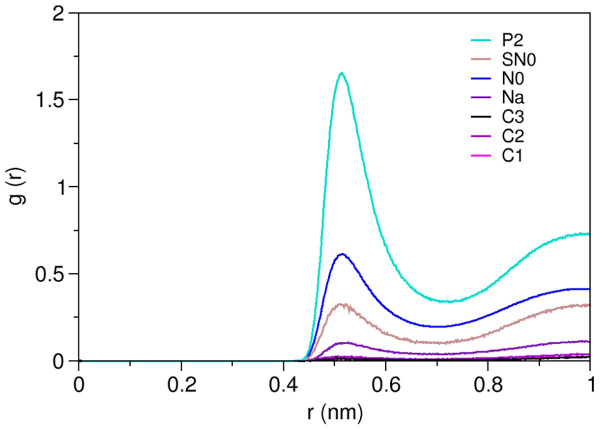

Figure S3. Average radial pair distribution functions for different CG particle types against water particles for 60 P80 molecules in the micellar configuration.

The lateral diffusion constant of the micelle was estimated to be 2.3 × 10^{-7} cm^2/s^{-1}, approaching well to the experimental value of 3.0 × 10^{-7} cm^2/s^{-1} [1], with a considerable improvement relative to the value of 1.8 × 10^{-7} cm^2/s^{-1} obtained in the previous course-grain modeling [2].

1.3. Effect of Surfactant Concentration on Micelle Size and Morphology

Experimental and *in silico* studies have shown that the surfactant micellar assemblies typically undergo size and morphological transition in response to the altered concentration [3–5]. The geometrical properties of surfactant carriers may impact their drug loading capacity and delivery efficiency [6,7]. The potential effect of surfactant concentration on the geometrical properties of the P80 micelle was examined by the stepwise increasing of concentration from 10 to 40 w% in a system of 60 surfactant molecules in water (main manuscript, Table 1, Simulations 2 and 5–7). Within the concentration range of 10–30 w%, semi-spherical micelles with no obvious morphological transition were formed. As the surfactant concentration exceeded 30 w%, the micelle begun to exhibit shape transition towards formation of a rod-like structure (Figure S4). Concentrations higher than 40 w%

were not considered to maintain the water sufficiently away from solvent phase reversal threshold. The sphere-to-cylinder morphological transition of micelles with the increase of surfactant concentration has been reported experimentally in several non-ionic surfactant systems [3,4], showing the consistency of our simulation results. The bulk concentration of surfactant above which the geometrical transition occurs is referred to as the second critical micelles concentration (SCMC) [4]. Therefore, based on our simulation data, a SCMC of 30 w% is attributable to P80.

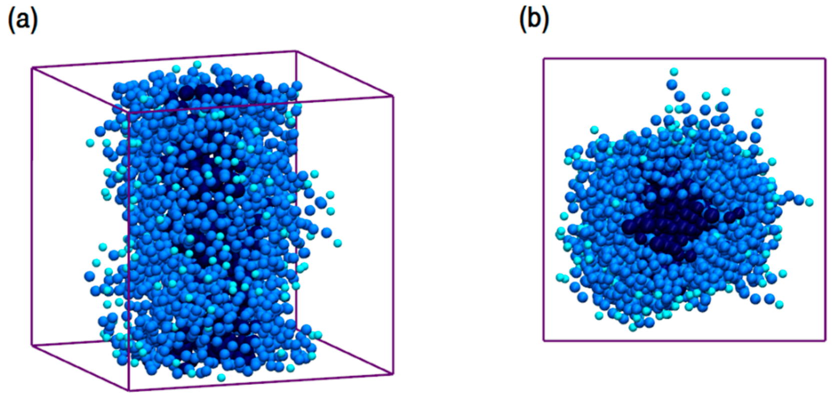

Figure S4. (**a**) 3D view and (**b**) cross-sectional view of the rod-like morphology of P80 aggregate at the surfactant concentration of 35 w%.

To examine the response of micelle size to surfactant concentration, a larger system size is required to allow formation of multiple clusters. Using 200 P80 molecules, a series of simulation setups was carried out at concentrations 10, 20, and 30 w% (main manuscript, Table 1, Simulations 8–10). To consider the possible effect of system initial configuration, each simulation was run 5 times at different initial random molecular distributions. The typical results of simulations at each of the above concentrations are presented in Figure S5. At 10 w%, the surfactant molecules are clustered to 6 aggregates, with the largest aggregate size of 62. Repeating the simulation at this concentration produced largest aggregate size of 56–65, which is similar to the N_{ag} of micelle used at smaller system size (see above). This, both verify the assumed aggregation number of P80 (60) in this study and shows the robustness of the simulated micellar system against system size, at least within the simulation timeframe. As seen from Figure S5, concentration rise to higher values is followed by formation of increasingly fewer clusters with larger average size. The direct dependence of micelle size on surfactant concentration has been observed consistently in various micellar system characterization studies, particularly in non-ionic surfactant solutions [3,4,8], showing the correlation of our simulation results with reality.

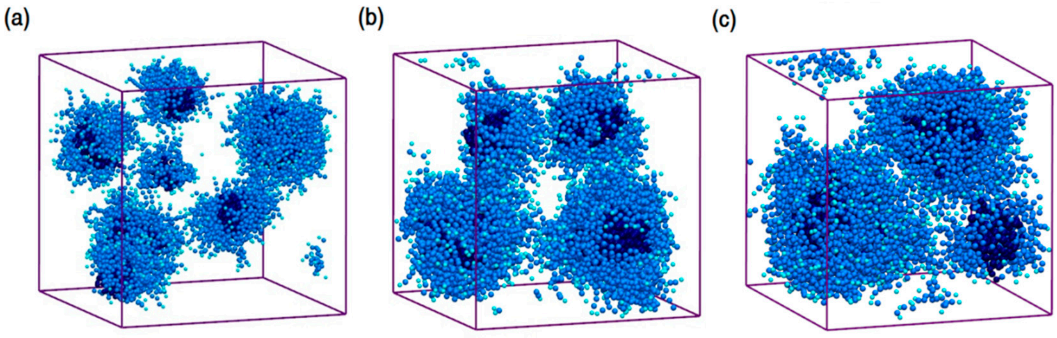

Figure S5. Size distribution of P80 micelles at concentrations (**a**) 10 w%, (**b**) 20 w%, and (**c**) 30 w%.

2. Simulation of PAs

Aggregation of PAs in the Aqueous Solution

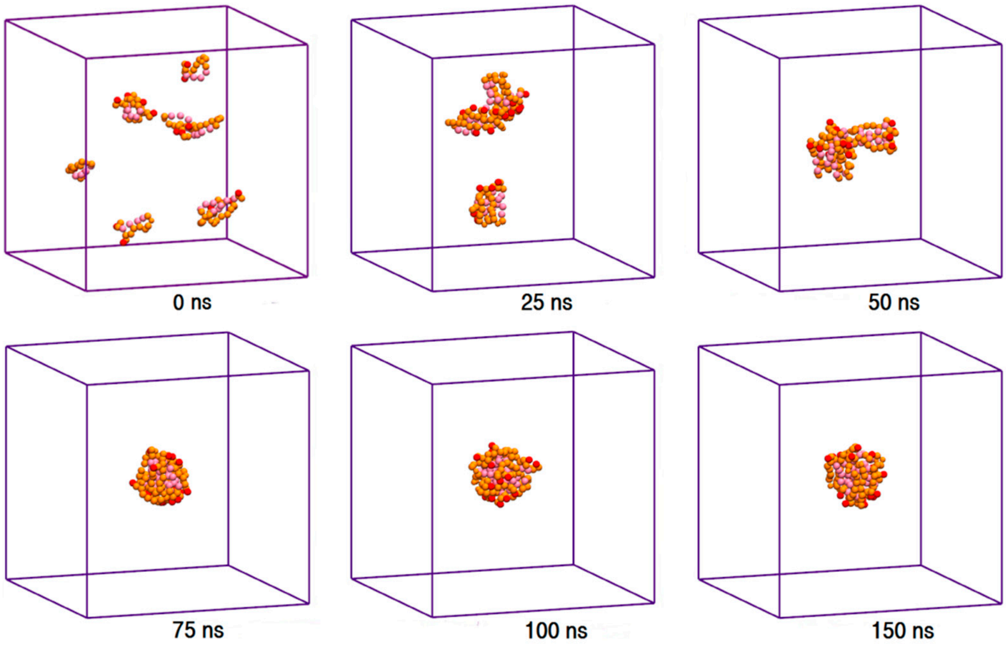

Figure S6. Trajectory snapshots of aggregation of Nys in the aqueous medium at different simulation times (water particles have been removed for clarity).

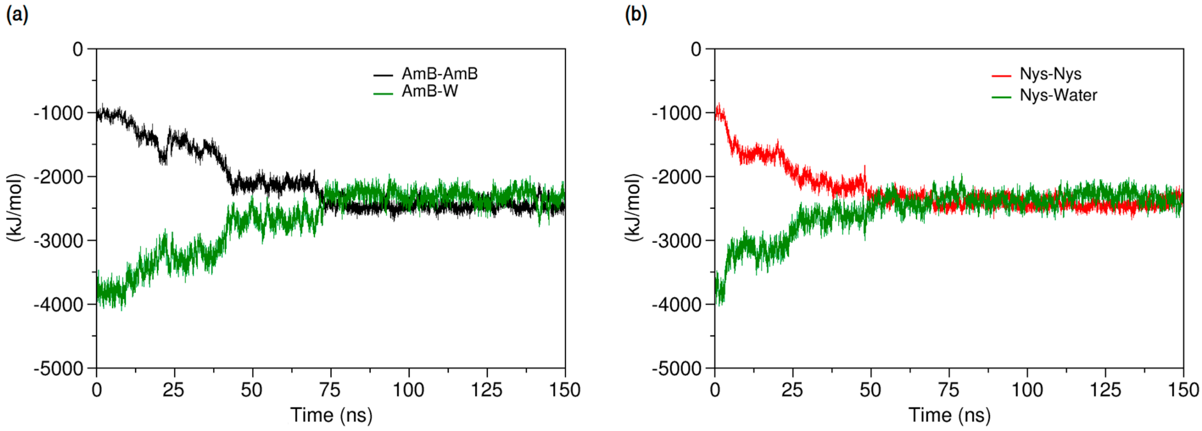

Figure S7. Time-evolution of the average energy of (**a**) AmB-AmB and AmB-W interactions and (**b**) Nys-Nys and Nys-W interactions during aggregation of PAs.

3. Simulation of P80-PA System

3.1. Interaction of PAs with a Single P80 Molecule

P80 has a four-branch hydrophilic POE chain, three of which terminated with CH2OH groups and one connected to the hydrocarbon chain. We were interested to identify whether any of these four branches are preferentially selected by PAs for interaction. In doing so, we calculated the RDF for each of the POE branches (main manuscript, Figure 15b; branch 1: 7–11, branch 2: 12–16, branch 3: 18, 21–24, and branch 4: 20, 26–29) against single PA molecules. As seen in Figure S8, while population of interaction sites of branches 2 to 4 around PAs are virtually similar, branch 1 interaction sites are nontrivially more concentrated around the drug molecules. Monitoring the trajectory made apparent that residence of PAs at the vicinity of the branch 1 encourages folding of the three other POE branches on PA molecules, thereby shielding them collectively against water, a

situation not readily accessible otherwise. Hence, more protection against water would be the reason for preferred interaction of PAs with branch 1. Such a positioning also provides more opportunity for the hydrocarbon chain to interact with PAs as reported in the main manuscript Section 2.3.1.

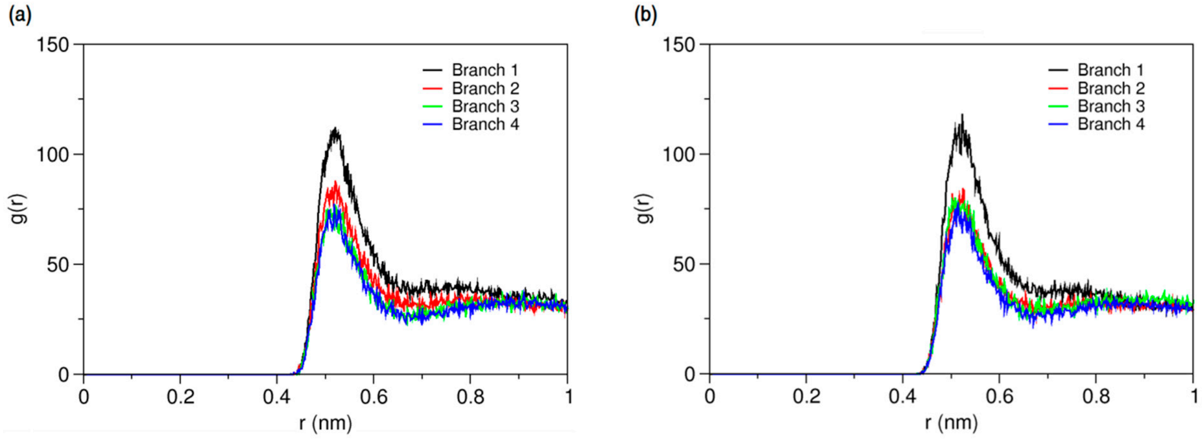

Figure S8. Average radial pair distribution functions for various POE branches of P80 against (**a**) AmB and (**b**) Nys.

3.2. Encapsulation of PAs into P80 Micelle

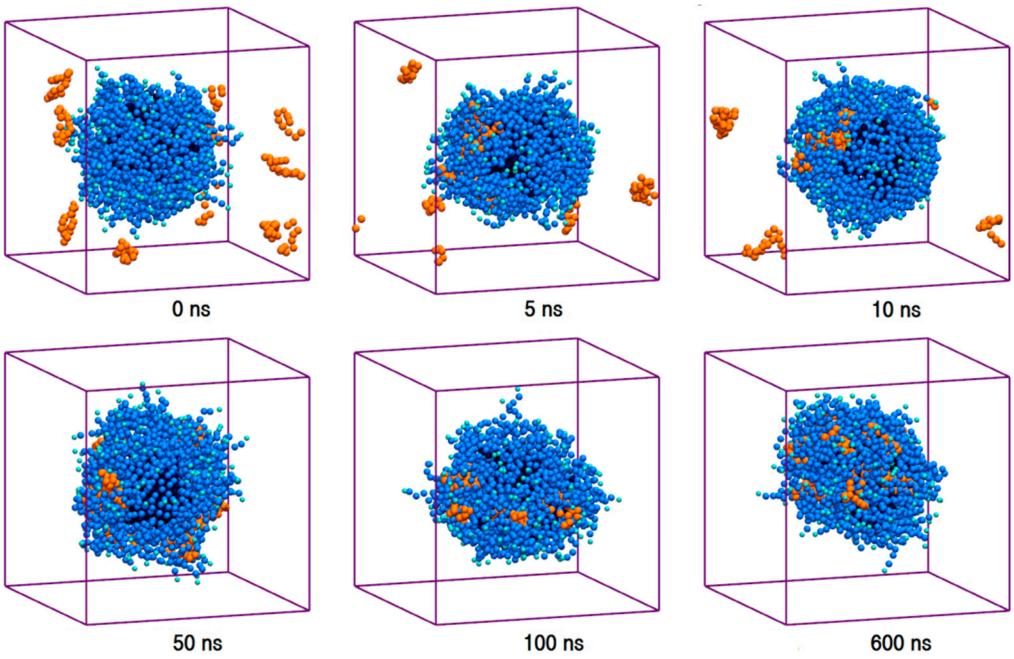

Figure S9. The trajectory snapshots of encapsulation of Nys molecules into P80 micelle at different simulation times (water particles have been removed for clarity).

3.3. Complementary Analysis of Properties of P80-PA Micellar Systems

3.3.1. Effect of PAs on the Conformational Properties of P80 Micelles

Micelles of amphiphilic carriers often undergo size and conformational transition upon encapsulating the hydrophobic drug molecules. Geometrical properties of drug-laden micelles may impact their loading capacity, delivery efficiency, therapeutic efficacy, and cytotoxicity [6,7]. To follow the possible PA-induced structural transition effect in P80, the $P80_{60}$-AmB-W and $P80_{60}$-Nys-W setups were extended to drug-to-surfactant ratios of 0.2 to 0.5 (main manuscript, Table 1, Simulations 17–21 and 29–33, respectively). The geometrical properties of P80 micelle loaded with various amounts of

each PA were investigated by calculating the R_g, SASA and PMI. We calculated R_g and SASA *vs.* drug-to-surfactant ratio for the whole micelle and the micelle core separately, to gain a more detailed picture of the potential drug-induced conformational changes. The increased loading of AmB was followed by linear increase of R_g (β = 0.243, R^2 = 0.98) and SASA (β = 0.308, R^2 = 0.99) of the whole micelle. The negligibility of drug loading dependence of R_g (β = −0.001, R^2 = 0.97) and SASA (β = −0.003, R^2 = 0.94) compared with that of the whole micelle indicates that only the micelle shell is expanded upon drug loading. Nearly identical results were also observed in corresponding $P80_{60}$-Nys-W systems. Nonetheless, the geometrical variation of the micelle shell is also small showing no dramatic drug-induced changes in size or integrity of the micelle, even at drug overload.

At the same time, the eccentricity values calculated from PMI revealed a linear but negligible increase in the mixed micelle sphericity (β = −0.001, R^2 = 0.99) with the PA load increment. Very similar results were obtained in the corresponding $P80_{60}$-Nys-W systems. This contrasts with the sphere-to-cylinder morphological transition upon drug load augment in the polymeric surfactant systems [9–11]. These data together with minor variation R_g and SASA with drug-to-surfactant ratio confirms the conformational robustness of P80 micelle against PA load.

3.3.2. Effect of Drug Load and Micelle Size on Localization of PAs

We further investigated the possible dependence of localization of AmB and Nys on drug load and micelle size. The effect of drug load was explored by analyzing the trajectories of simulations performed at drug-to-surfactant ratios of 0.1 to 0.5 (main manuscript, Table 1, Simulations 17–21 and Simulations 29–33, respectively). The ratio of drug-locus-to-micelle-radius (R_d/R_m) for both drugs showed a linear but little dependence on drug-to-surfactant ratio (β = 0.002, R^2 = 0.99). The effect of micelle size was pursued by introducing a fixed number of each of AmB and Nys molecules (9) to the micelles of 60, 100, and 150 P80 surfactant molecules (main manuscript, Table 1, Simulations 17, 22, and 23 and Simulations 29, 34, 35, respectively). The results again showed a very small variation of drugs placement with the micelle size for both drugs (β = 0.0003, R^2 = 0.97). Accordingly, the impact of drug load and micelle size on localization of PAs is concluded to be negligible.

3.3.3. Effect of PAs on the Stability of P80 Micellar System

The effect of PAs on micelle-micelle interactions and the stability of the mixed micelles form was also examined. A fixed concentration (1 w%) of each of Amb and Nys was separately dispersed in the solutions of 10, 20, and 30 w% of surfactant. The simulations were carried out in the equilibrated systems of 200 P80 molecules clustered into micellar aggregates of various sizes (main manuscript, Table 1, Simulations 24–26 and Simulations 36–38, respectively), depending on surfactant concentration. In all systems, encapsulation of the introduced drug molecules was accompanied by fusion of two or more neighboring micelles, and thereby increased size of clusters (Figure S10). Particularly, at the surfactant concentration of 30 w%, introduction of PAs resulted in the collapse of all micelles into a single large micellar aggregate. Therefore, the stability of P80 micellar system is concluded to be sensitive to introduction of PAs, even at moderate drug loads and surfactant concentrations. While controlled growth of micelle size may improve the drug loading capacity/efficiency as a result of increased solubilization space [12,13], the drug-induced excessive micelle fusions may lead to formation of very large mixed surfactant-drug aggregates, and thereby increase the probability of formulation precipitation. Therefore, a delicate balance between drug loading and micelle growth, which may ultimately pose a constraint on drug loading efficiency, should be regarded for stability of the mixed micellar system [12]. The increased size of micelles upon introduction of hydrophobic drugs has also been observed in other micellar systems including polymeric [9–11] and detergent [14] surfactant solutions.

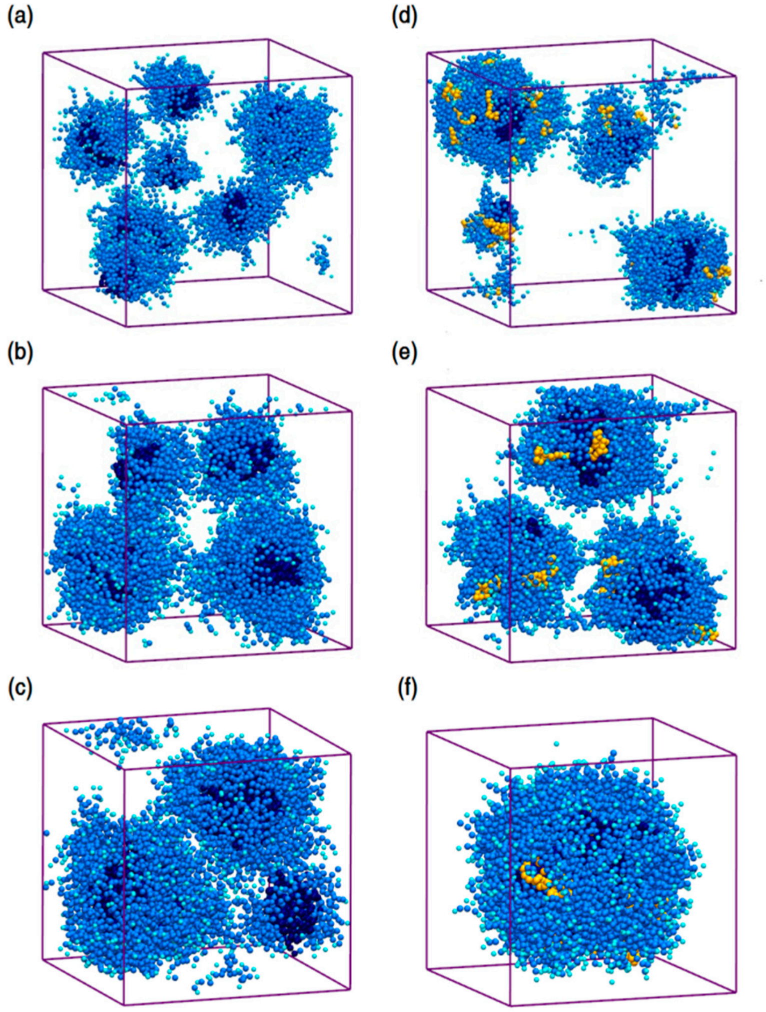

Figure S10. (**a**–**c**): Equilibrium size distribution of P80 micelles at the surfactant concentrations of 10, 20, and 30 w%, respectively. (**d**–**f**): Equilibrium size distribution of P80 micelles at the surfactant concentrations of 10, 20, and 30 w%, respectively, after the introduction of 1 w% AmB.

3.3.4. Pattern of PAs Distribution in Micellar Solution

We also monitored the loading distribution of PAs among in multi-micellar system. Figure S11 compares the density profile of P80-AmB nanoparticles in a sample simulated system containing 1 w% of AmB and 10 w% of P80, at the drug-to-surfactant ratio of 10% (Table 1, Simulation 24). As seen, while the largest micelle has accommodated the highest amount of drug, the second largest micelle and the smallest aggregate have loaded similar amounts, whereas the third largest micelle has remained relatively empty. This heterogeneous distribution of drug was typical of our other simulations for both AmB and Nys at various initial system setups. Although lack of direct experimental data makes the comparison difficult, our results are consistent with the experimentally observed heterogeneous distribution of AmB in sodium deoxycholate (SDC) micellar solution, in which the SDC-AmB mixed micelles were found to coexsist with drug free micelles [15,16]. Similar drug distribution pattern was also reported experimentally and *in silico* in the micellar system of GCPQ loaded with propofol in the work of Ahmed *et al.* [17]. Non-homogenous distribution of drug in micellar solution may negatively impact the solubilization efficiency, because while some drug-laden micelles may become overloaded some others may remain unused. From the simulation trajectories it was inferred that the distance of PA molecules from neighboring micelles rather than the micelle size preliminary influences the distribution of drugs. In other words, the drug molecules tend to migrate from the bulk to the nearest neighboring micelle, regardless of its size. In addition,

the fused micelles which were formed upon introduction of drug were found typically more loaded than the intact micelles.

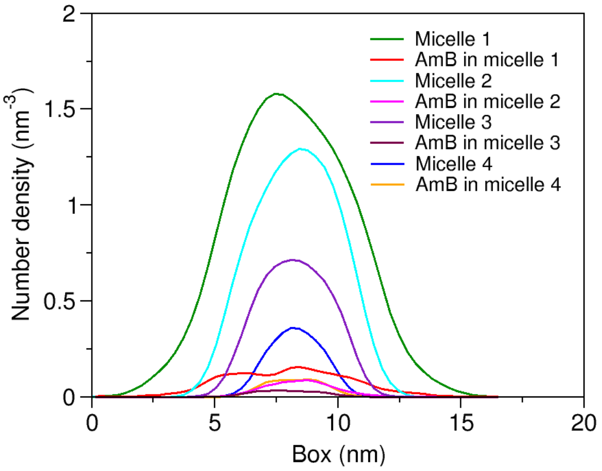

Figure S11. Density profile for four AmB loaded P80 micelles in a multi-micellar system containing 200 P80 molecules (10 w%).

3.3.5. Drug Loading Capacity

The theoretical drug loading capacity of a surfactant shows the ideal room for improving its solubilization efficiency, hence is of relevance for formulation practice. As seen above, P80 is structurally robust against PAs even at high overloads. Therefore, it is difficult to determine the maximum theoretical loading capacity of this surfactant for the PAs, based on structural stability. However, regarding the importance of PAs aggregation state in their formulation, we considered the theoretical loading of P80 as its maximum capacity for encapsulating these drug molecules in monomeric form. Profiling SASA at various drug-to-surfactant ratios revealed signals of aggregate formation in the micelle at ratio of above 30 w%. Based on the above definition, hence, an optimal theoretical loading of 23 w% (correspond to drug-to-surfactant ratio of 30%) is determined for P80 as a carrier of AmB or a Nys.

4. Experimental Section

4.1. Modeling of P80 and PAs

The coarse-grained mapping of P80 molecular structure is presented in Figure 15a (main manuscript). P80 was modeled by 30 CG sites, 24 of which representing the hydrophilic head group, and 6 representing the hydrophobic tail. A combination of 4-to-1 (C1/C3 particle types) and 3-to-1 (C2 particle type) mapping was adopted for representation of the alkyl chain. An ethylene oxide (EO) chain identical to the experimentally determined EO chain of Polysorbate 60 [18] with similar distribution was assumed for P80. The C–O–C=O group of the hydrophilic head was represented as a CG particle of Na type. The terminal polar group, C–O–H, was represented by the CG particle of P2 type. The ring on the head group was mapped with a triangular configuration to three SN0 particles following a 2 to 1 mapping approach. All other hydrophilic molecular groups were modelled by 3 to 1 mapping to N0 particle type. The model was initially parameterized according to the default MARTINI force field parameters and optimized to reproduce the structural properties of the corresponding united-atom model, including distances, angles, and dihedrals. An aggregation number (N_{ag}) of 60 was attributed to the P80 micelle based description of Tween 80 product by the manufacturers [19,20]. The CG model was validated by comparing the radius of gyration (R_g), eccentricity (e), and solvent accessible surface area (SASA) of the CG micelle with the corresponding values reported from atomic-scale simulation of the Tween 80 micelle of canonical structure with

equal number of EO unites in each of the four head groups at N_{ag} of 60 [21] and by reproduction of N_{ag} and R_g of the largest micellar clusters at different system sizes.

The coarse-grained mapping of AmB and Nys molecular structures is illustrated in Figure 15b,c (main manuscript), respectively. PAs were represented by 17 CG sites. The hydrophobic polyene chain was modeled by the C3 and C4 particle types using a 4 to 1 mapping. The hydroxyl groups including the ring were modeled by P-type particles. The mycosamine ring was represented using 2 to 1 mapping approach, based on the MARTINI representation of ring structures [22]. Thereby, the ring was modeled as three beads in triangular configuration, including a positively charged Qd_a particle representing the aminated and hydroxylated groups, and two particles of SN0 and SP1 types. The carboxyl moiety was modeled as a negatively charged Qd_a particle type following a 4 to 1 mapping. The whole macrolactone ring, hence, is modeled as a CG molecule with net zero charge. The CG models of PAs were initially parameterized according to the standard MARTINI force filed. The models were further optimized by fitting the structural properties of the CG models, to those of the corresponding united-atom models.

4.2. Simulation

MD simulations were carried out using GROMACS v. 4.6.3 simulation package [23] and the simulation trajectories were visualized using VMD [24]. The equation of motion was integrated by leap frog algorithm [25] with a time step of 30 fm. The cutoff for the nonbonded interactions was set to r_{cut} = 1.2 nm. The Lennard–Jones (LJ) potential was shifted from 0.9 nm to r_{cut}, and the Coulomb potential was shifted from 0 to r_{cut}. All simulations were carried out in NPT ensemble at 298 K and 1 bar with periodic boundary conditions for cubic boxes. The temperature and pressure were maintained isotropically using the Berendsen barostat and thermostat [26] with a coupling time constant of 1.5 ps and 3 ps, respectively. The 4-to-1 CG representation of water validated by Marrink *et al.* [27] was adopted as the solvent. To prevent freezing of CG water at simulation temperature (298 K), approximately 10% of water was substituted by antifreeze particles [22] in all simulations. The mass of all CG particles was considered the same as the mass of CG water particle, *i.e.*, 72 amu, with the exception of the ring species which took a mass of 45 amu [22].

Multiple time frames were considered for various stages of the study. Single P80, single AmB, and their single molecular interactions as well as self-aggregation of PAs were simulated over a 150-ns time frame. A time frame of 600 ns was specified for P80 self-aggregation and encapsulation of PAs into the micelle (main manuscript, Table 1). Of the simulation times of 150 and 600 ns, the first 20 and 100 ns, respectively, was considered for equilibration phase, and the rest was used for analysis. The equilibrium state was determined by monitoring the root mean square distance (RMSD), potential energy, and R_g.

4.3. Analysis

The hydration and relative distribution of the molecules and their interaction sites were calculated by radial distribution function (RDF) analysis [28]. The morphology of molecular aggregates were characterized based on principal moments of inertia (PMI) with the semi-axis order of I1 > I2 > I3. The solvent accessible surface area (SASA) was calculated by using a probe molecule with half a radius of the CG water particle (2.35 Å). The diffusion constant of molecules was estimated using Einstein equation $d = \langle \Delta r^2 \rangle / 2nt$, where $\langle \Delta r^2 \rangle$ represents average mean square displacement (MSD) over the time period of t , and n denotes the number dimensions. Drug-to-surfactant ration was defined as the total weight of drug divided by total weight of surfactant. The drug load was defined as the total weight of the drug divided by the total weight of drug and surfactant.

References

1. Verbrugghe, M.; Cocquyt, E.; Saveyn, P.; Sabatino, P.; Sinnaeve, D.; Martins, J.C.; Van der Meeren, P. Quantification of hydrophilic ethoxylates in polysorbate surfactants using diffusion NMR spectroscopy. *J. Pharm Biomed. Anal.* **2010**, 51583–51589.
2. Amani, A.; York, P.; de Waard, H.; Anwar, J. Molecular dynamics simulation of a polysorbate 80 micelle in water. *Soft Matter* **2011**, 72900–72908.
3. Velinova, M.; Sengupta, D.; Tadjer, A.V.; Marrink, S.-J. Sphere-to-rod transitions of nonionic surfactant micelles in aqueous solution modeled by molecular dynamics simulations. *Langmuir* **2011**, *27*, 14071–14077.
4. Al-Anber, Z.A.; i Avalos, J.B.; Floriano, M.A.; Mackie, A.D. Sphere-to-rod transitions of micelles in model nonionic surfactant solutions. *J. Chem. Phys.* **2003**, 1183816–113826.
5. Rosen, M.J. *Surfactants and Interfacial Phenomena*, 2nd ed.; Wiley-Interscience: New York, NY, USA, 1989; Volume 1, pp. 108–143.
6. Loverde, S.M.; Klein, M.L.; Discher, D.E. Nanoparticle shape improves delivery: Rational coarse grain molecular dynamics (rCG-MD) of taxol in worm-like PEG-PCL micelles. *Adv. Mater.* **2012**, *24*, 3823–3830.
7. Christian, D.A.; Cai, S.; Garbuzenko, O.B.; Harada, T.; Zajac, A.L.; Minko, T.; Discher, D.E. Flexible filaments for *in vivo* imaging and delivery: Persistent Circulation of filomicelles opens the dosage window for sustained tumor shrinkage. *Mol. Pharm.* **2009**, *6*, 1343–1352.
8. Shinoda, W.; DeVane, R.; Klein, M.L. Coarse-grained molecular modeling of non-ionic surfactant self-assembly. *Soft Matter* **2008**, *4*, 2454–2462.
9. Luo, Z.; Jiang, J. pH-sensitive drug loading/releasing in amphiphilic copolymer PAE–PEG: Integrating molecular dynamics and dissipative particle dynamics simulations. *J. Control. Release* **2012**, *162*, 185–193.
10. Giacomelli, C.; Schmidt, V.; Borsali, R. Specific interactions improve the loading capacity of block copolymer micelles in aqueous media. *Langmuir* **2007**, *23*, 6947–6955.
11. Nagarajan, R. Solubilization of hydrocarbons and resulting aggregate shape transitions in aqueous solutions of Pluronic® (PEO–PPO–PEO) block copolymers. *Colloids Surf. B Biointerfaces* **1999**, *16*, 55–72.
12. Guo, X.D.; Qian, Y.; Zhang, C.Y.; Nie, S.Y.; Zhang, L.J. Can drug molecules diffuse into the core of micelles? *Soft Matter* **2012**, 8, 9989–9995.
13. Shuai, X.; Ai, H.; Nasongkla, N.; Kim, S.; Gao, J. Micellar carriers based on block copolymers of poly(ε-caprolactone) and poly(ethylene glycol) for doxorubicin delivery. *J. Control. Release* **2004**, 98415–98426.
14. Dignam, J.D.; Qu, X.; Ren, J.; Chaires, J.B. Daunomycin binding to detergent micelles: A model system for evaluating the hydrophobic contribution to drug−DNA interactions. *J. Phys. Chem. B* **2007**, *111*, 11576–11584.
15. Lamy-Freund, M.T.; Ferreira, V.F.N.; Schreier, S. Polydispersity of aggregates formed by the polyene antibiotic amphotericin B and deoxycholate. A spin label study. *Biochim. Biophys. Acta (BBA)—Biomembr.* **1989**, *981*, 207–212.
16. Lamy-Freund, M.; Schreier, S.; Peitzsch, R.; Reed, W. Characterization and time dependence of amphotericin B: deoxycholate aggregation by quasielastic light scattering. *J. Pharm Sci.* **1991**, *80*, 262–266.
17. Ahmad, S.; Johnston, B.F.; Mackay, S.P.; Schatzlein, A.G.; Gellert, P.; Sengupta, D.; Uchegbu, I.F. *In silico* modelling of drug–polymer interactions for pharmaceutical formulations. *J. R. Soc. Interface* **2010**, *7*, S423–S433.
18. Vu Dang, H.; Gray, A.I.; Watson, D.; Bates, C.D.; Scholes, P.; Eccleston, G.M. Composition analysis of two batches of polysorbate 60 using MS and NMR techniques. *J. Pharm Biomed. Anal.* **2006**, *40*, 1155–1165.
19. TWEEN® 80. Available online: http://www.sigmaaldrich.com/content/dam/sigma-aldrich/docs/Sigma/Instructions/detergent_selection_table.pdf (accessed on 5 October 2015).
20. Tween™ 80 Surfact-Amps™ Detergent Solution. Available online: http://www.piercenet.com/product/tween-80-detergentsolution (accessed on 5 October 2015).
21. Tang, X.; Huston, K.J.; Larson, R.G. Molecular dynamics simulations of structure–property relationships of tween 80 surfactants in water and at interfaces. *J. Phys. Chem. B* **2014**, *118*, 12907–12918.
22. Marrink, S.J.; Risselada, H.J.; Yefimov, S.; Tieleman, D.P.; de Vries, A.H. The MARTINI force field: Coarse grained model for biomolecular simulations. *J. Phys. Chem. B* **2007**, *111*, 7812–7824.
23. Berendsen, H.J.C.; van der Spoel, D.; van Drunen, R. GROMACS: A message-passing parallel molecular dynamics implementation. *Comput. Phys. Commun.* **1995**, *9*, 143–156.
24. Humphrey, W.; Dalke, A.; Schulten, K. VMD: Visual molecular dynamics. *J. Mol. Graph.* **1996**, 14, 33–38.
25. Allen, M.; Tildesley, D. *Computer Simulation of Liquids*; Oxford University Press: Oxford, UK, 1989; pp. 71–84.

26. Berendsen, H.J.C.; Postma, J.P.M.; van Gunsteren, W.F.; di Nola, A.; Haak, R.J. Molecular dynamics with coupling to an external bath. *J. Chem. Phys.* **1984**, *81*, 3684–3690.
27. Marrink, S.J.; de Vries, A.H.; Mark, A.E. Coarse grained model for semiquantitative lipid simulations. *J. Phys. Chem. B* **2003**, *108*, 750–760.
28. Allen, M.; Tildesley, D. *Computer Simulation of Liquids*; Oxford University Press: Oxford, UK, 1989; pp. 182–211.
